# Human MC4R variants affect endocytosis, trafficking and dimerization revealing multiple cellular mechanisms involved in weight regulation

**DOI:** 10.1016/j.celrep.2021.108862

**Published:** 2021-03-23

**Authors:** Bas Brouwers, Edson Mendes de Oliveira, Maria Marti-Solano, Fabiola B.F. Monteiro, Suli-Anne Laurin, Julia M. Keogh, Elana Henning, Rebecca Bounds, Carole A. Daly, Shane Houston, Vikram Ayinampudi, Natalia Wasiluk, David Clarke, Bianca Plouffe, Michel Bouvier, M. Madan Babu, I. Sadaf Farooqi, Jacek Mokrosiński

**Affiliations:** 1 University of Cambridge Metabolic Research Laboratories and NIHR Cambridge Biomedical Research Centre, Wellcome-MRC Institute of Metabolic Science, Addenbrooke’s Hospital, Cambridge CB2 0QQ, UK; 2 MRC Laboratory of Molecular Biology, Cambridge, UK; 3 Institute for Research in Immunology and Cancer, Department of Biochemistry and Molecular Medicine, Université de Montréal, Montréal, QC H3T 1J4, Canada; 4 Wellcome-Wolfson Institute for Experimental Medicine, School of Medicine, Dentistry and Biomedical Sciences, Queen’s University Belfast, Belfast BT9 7BL, UK; 5 Department of Structural Biology and Center for Data Driven Discovery, St Jude Children’s Research Hospital, Memphis, TN, USA

**Keywords:** obesity, MC4R, therapy, weight loss, GPCRs, β-arrestin, Gα_s_, melanocortin, MSH

## Abstract

The Melanocortin-4 Receptor (MC4R) plays a pivotal role in energy homeostasis. We used human *MC4R* mutations associated with an increased or decreased risk of obesity to dissect mechanisms that regulate MC4R function. Most obesity-associated mutations impair trafficking to the plasma membrane (PM), whereas obesity-protecting mutations either accelerate recycling to the PM or decrease internalization, resulting in enhanced signaling. MC4R mutations that do not affect canonical Gα_s_ protein-mediated signaling, previously considered to be non-pathogenic, nonetheless disrupt agonist-induced internalization, β-arrestin recruitment, and/or coupling to Gα_s_, establishing their causal role in severe obesity. Structural mapping reveals ligand-accessible sites by which MC4R couples to effectors and residues involved in the homodimerization of MC4R, which is disrupted by multiple obesity-associated mutations. Human genetic studies reveal that endocytosis, intracellular trafficking, and homodimerization regulate MC4R function to a level that is physiologically relevant, supporting the development of chaperones, agonists, and allosteric modulators of MC4R for weight loss therapy.

## Highlights

• Obesity-associated MC4R mutations that do not reduce cAMP disrupt other processes
• MC4R mutations impact receptor homodimerization, endocytosis, and trafficking
• Obesity-protecting mutations increase plasma membrane MC4Rs and enhance signaling
• Multiple mechanisms regulate melanocortin tone to a physiologically relevant level

## Graphical abstract

**Figure:**
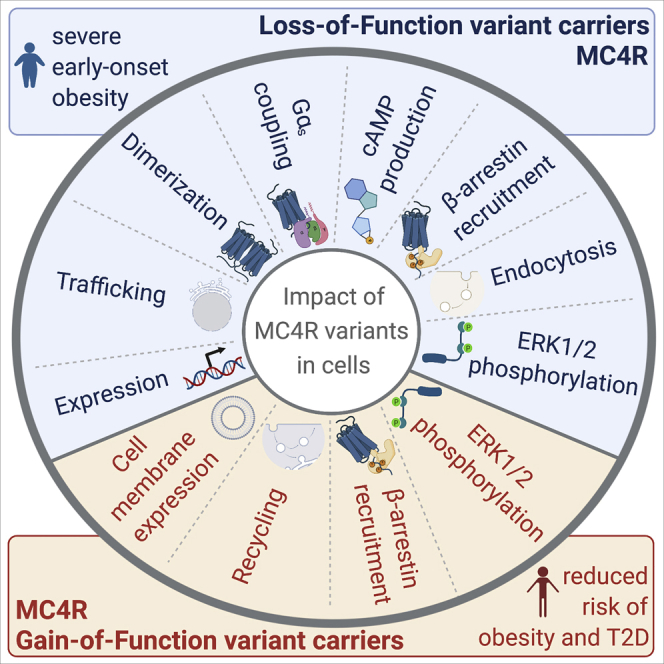

## Teaser

Using mutations in the human Melanocortin-4 Receptor (MC4R), Brouwers et al. identify receptor trafficking and endocytosis, coupling to Gα_s_/β-arrestins, and homodimerization as mechanisms involved in the regulation of body weight that may be targeted for weight loss therapy.

## Introduction

Obesity-associated metabolic complications such as type 2 diabetes and cardiovascular disease contribute to significant morbidity, mortality, and health care costs (Heymsfield and Wadden, 2017). As such, there is a substantial unmet need for safe and effective weight loss therapies. G-protein-coupled receptors (GPCRs) are the largest family of cell surface proteins involved in signal transduction (Hauser et al., 2017) and are targeted by more than 30% of prescribed medicines. In the last decade, new insights into GPCR pharmacology and structure-function relationships have paved the way for the development of compounds with diverse mechanisms of action, increasing treatment options in the clinic.

Here, we focused on the brain-expressed Melanocortin-4 Receptor (MC4R), a GPCR that plays a pivotal role in weight regulation and is a major target for drug development. With multiple potentially druggable sites accessible at the cell surface (Hauser et al., 2017) and informed by the recently published 3D structure of MC4R (Yu et al., 2020) that provides a template for structure-based drug discovery, it is timely to investigate new opportunities to target MC4R for weight loss therapy. It is well-established that binding of Pro-opiomelanocortin (POMC)-derived peptides (α-/β-MSH [melanocyte-stimulating hormone]) to membrane-bound MC4R activates G proteins (Gα_s_) and stimulates the production of cyclic AMP (cAMP) to reduce food intake in the fed state (Schwartz et al., 2000). In classical models of GPCR activation, G-protein-dependent signaling is regulated by intracellular scaffolding proteins, β-arrestins, whose interaction with the receptor drives its endocytosis and targeting to early endosomes (EEs) followed by subsequent recycling to the plasma membrane (PM) or sorting to lysosomes for degradation (Shinyama et al., 2003). Endocytosis of GPCRs also contributes to mitogen-activated protein kinase (MAPK) pathway activation, facilitating a second wave of signaling and gene transcription by Extracellular signal-regulated kinase 1/2 (ERK1/2) phosphorylation (Rajagopal and Shenoy, 2018).

Quantitative variation in MC4R signaling plays a pivotal role in energy homeostasis (Huszar et al., 1997; Vaisse et al., 1998; Yeo et al., 1998), as the loss of one *MC4R* allele is sufficient to cause severe obesity in rodents and humans. Additionally, heterozygous mutations in MC4R that impair cAMP production represent the commonest monogenic form of obesity (Farooqi et al., 2003). However, up to 25% of MC4R mutations found in obese cohorts do not reduce cAMP production and are therefore classified as wild-type (WT)-like/non-pathogenic (https://www.mc4r.org.uk; Collet et al., 2017). Here, we set out to establish a deeper understanding of the mechanisms that control MC4R function to inform new strategies for drug development (Sharma et al., 2019; Tao, 2010; Yang and Tao, 2017). As therapeutic targets supported by human genetic evidence are more likely to succeed through the drug discovery pipeline (Nelson et al., 2015), we used naturally occurring missense variants in *MC4R* as tools to investigate the impact of disruption of specific mechanisms on the regulation of body weight in humans.

## Results

**Figure 1:**
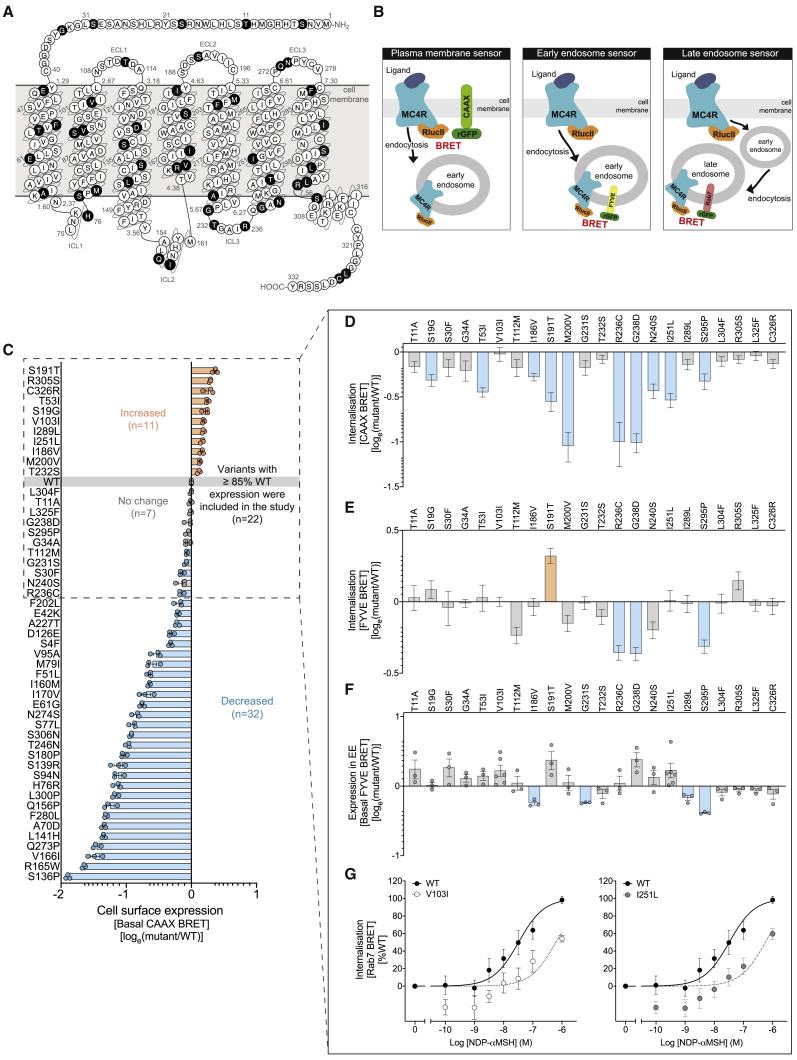
MC4R mutants impair PM localization and agonist-dependent endocytosis (A) Schematic of human MC4R. Amino acids affected by obesity-associated and obesity-protecting MC4R mutants are indicated in black (n = 50). Sequence-based generic GPCR residue numbering for Family A GPCRs (Ballesteros-Weinstein) is included. ECL, extracellular loop; ICL, intracellular loop. (B) Agonist-induced MC4R internalization is quantified using ebBRET-based sensors expressed at either the plasma membrane (PM; rGFP-CAAX sensor), early endosomes (EEs; rGFP-FYVE sensor), or in late endosomes (LEs; drGFP-Rab7 sensor). Renilla luciferase II (RlucII) serves as the ebBRET donor and is coupled to MC4R. (C) Quantification of receptor levels at the PM by basal ebBRET signal. Twenty-two mutants show PM expression of ≥85% of WT and were taken forward for functional assays. (D and E) Agonist-induced MC4R internalization as quantified using the PM sensor (D) and the EE sensor (E), both normalized to basal ebBRET. (F) Basal ebBRET as a proxy of receptor level in EEs. (G) Agonist-induced internalization quantified using LE sensor for the gain-of-function (GoF) mutants V103I and I251L, normalized to basal ebBRET quantified using the PM sensor. Data in (C)–(F) are expressed as log_e_ (mutant/WT) and plotted as mean ± standard error from 3–6 independent experiments. Data in (D) and (E) represent mean ± standard error from the sum curves and therefore do not include data points from individual experiments. Data in (G) are expressed as % WT MC4R and plotted as mean ± standard error from 3 independent experiments. Mutants are classified as GoF (orange), LoF (blue), or WT-like (gray) based on statistically significant differences between WT and mutant (unpaired t test with Welch’s correction; p < 0.05, or extra sum-of-squares F test for D and E; p < 0.05). See also Figures S1–S3 and S6.

We characterized 48 rare *MC4R* variants (minor allele frequency [MAF], <1%) associated with severe obesity in clinically ascertained cohorts (Farooqi et al., 2003; Hinney et al., 2006; Stutzmann et al., 2008) and two prevalent *MC4R* variants (MAF, 1%–2%; V103I and I251L) associated with protection from obesity in large population-derived studies (Lotta et al., 2019; Stutzmann et al., 2007; Wang et al., 2010; Young et al., 2007; Figure 1A; Table S1). We preferentially selected variants that did not affect cAMP accumulation in previous studies (19/48) and used a range of assays to investigate control points that tightly regulate GPCR signaling. Experiments were conducted in HEK293 cells that do not endogenously express MC4R.

### MC4R mutations alter membrane expression, endocytosis, and trafficking

To quantitatively assess endocytosis and intracellular trafficking in live cells, we used enhanced bystander bioluminescence resonance energy transfer (ebBRET)-based sensors that rely on the transfer of energy between a bioluminescent donor, Renilla luciferase (RLuc), and a fluorescent acceptor, green fluorescent protein (Renilla GFP [rGFP]) (Namkung et al., 2016). By anchoring rGFP at the PM (rGFP-CAAX sensor) to early (EE; rGFP-FYVE sensor) and late endosomes (LEs; tandem rGFP [drGFP]-Rab7 sensor) (Figure 1B), we were able to quantify agonist-induced changes in MC4R expression in these cellular compartments in experiments conducted in HEK293 cells transiently transfected with WT or mutant forms of MC4R. We found that the majority of MC4R mutants tested reduced cell surface localization quantified with the PM sensor (basal ebBRET signal) (Figure 1C). Comparable results were obtained using an enzyme-linked immunosorbent assay (ELISA) and in radiolabeled ligand binding assays (Figures S1A–S1H). As one might predict, most MC4R mutants that decreased PM localization exhibited reduced agonist-promoted internalization (Figures S1I–S1K).

To investigate whether MC4R mutations that do not impair PM localization (defined as values ≥85% of WT) can disrupt receptor function, we focused on 22 mutants with normal or increased PM expression (Figure 1C). In BRET assays, agonist-induced internalization of WT MC4R occurs rapidly, reaching a maximum within 20 min (Figure S1L). We found that several MC4R mutants with normal or higher PM expression affected agonist-induced internalization; 10 variants reduced trafficking away from the PM, whereas 3 reduced and 1 increased trafficking to EEs (Figures 1D and 1E; Figures S2 and S3). Changes in endocytosis may impact both homeostatic control of receptor number at the cell surface and receptor signaling originating from endosomal compartments (Irannejad and von Zastrow, 2014). We found that some mutants showed reduced basal presence in EEs (n = 3; Figure 1F), which might reflect disrupted constitutive internalization (i.e., in absence of agonist), shown to be essential to maintain receptor responsiveness to α-MSH (McDaniel et al., 2012).

The obesity-protecting MC4R mutants (V103I and I251L) showed increased presence at the PM with normal (V103I) or reduced (I251L) agonist-induced internalization (Figures 1C and 1D). Using a sensor localizing to LEs/lysosomes, we observed reduced expression of V103I and I251L MC4R in this compartment (Figure 1G). As such, the increased presence of obesity-protecting MC4R mutants at the PM is likely to be explained by accelerated recycling to the PM for V103I MC4R and reduced internalization for I251L MC4R. Increased PM expression of these two mutants leads to increased exposure to extracellular ligands and enhanced intracellular signaling (gain of function), which explains the association between these variants and a substantial reduction in the risk of obesity and type 2 diabetes (Lotta et al., 2019).

**Figure 2:**
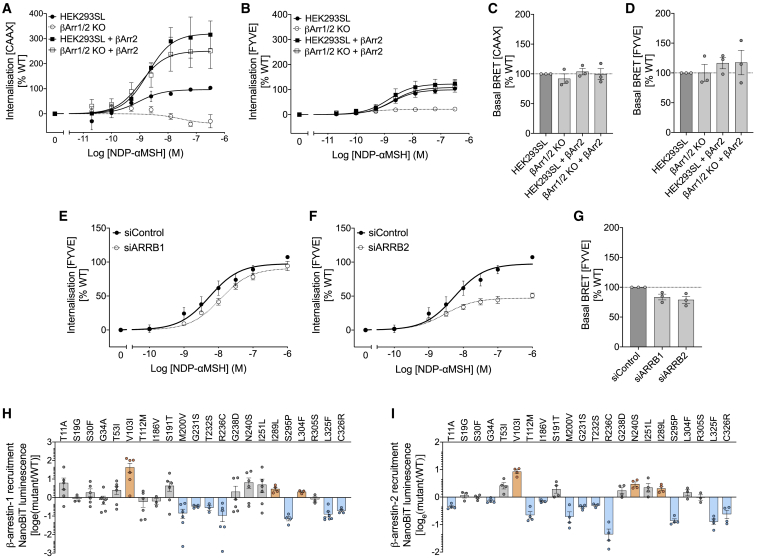
β-arrestin-2 drives MC4R internalization, and obesity-associated MC4R mutants impair β-arrestin recruitment (A–D) Agonist-dependent endocytosis of MC4R in ARRB1/2 knockout and parental HEK293SL control cells transfected with a β-arrestin-2 expression vector, as measured using the PM sensor (A) or the EE sensor (B); basal ebBRET with the PM sensor (C) and the EE sensor (D) in this experiment. (E and F) Agonist-dependent endocytosis quantified in HEK293SL cells transfected with siRNAs targeting no mRNA (siControl) and β-arrestin-1 (siARRB1) (E) or β-arrestin-2 (siARRB2) (F). (G) Basal ebBRET in this experiment using siControl and siARRB1 or siARRB2. (H and I) Agonist-dependent β-arrestin-1 (H) and β-arrestin-2 (I) recruitment for MC4R mutants that do not impair receptor number at the PM. Internalization expressed as percentage of the maximal ΔebBRET obtained with WT receptor (see STAR Methods) and plotted as mean ± standard error from 3 independent experiments. β-arrestin recruitment data are expressed as log_e_ (mutant/WT) and plotted as mean ± standard error from 3–6 independent experiments. In (H) and (I), mutants are classified as GoF (orange), LoF (blue), or WT-like (gray) based on statistically significant differences between WT and mutant (unpaired t test with Welch’s correction; p < 0.05). See also Figure S4.

Here, we found that upon ligand stimulation, agonist-dependent internalization of WT MC4R, quantified as a change in BRET signal (using both PM and EE sensors), was impaired in cells lacking β-arrestin-1 and β-arrestin-2 (Figures 2A and 2B). Interestingly, there was no change in basal receptor expression at the PM defined by the basal BRET signal (Figures 2C and 2D), suggesting that constitutive internalization is β-arrestin-independent, as shown previously for MC4R (McDaniel et al., 2012) and other GPCRs (Paing et al., 2006). To further evaluate the contribution of β-arrestins to the internalization of MC4R, we performed small interfering RNA (siRNA)-mediated knockdown of β-arrestin-1 and -2 in HEK293 cells. Knockdown of β-arrestin-2 impaired agonist-dependent endocytosis of MC4R, whereas knockdown of β-arrestin-1 had a minimal effect in this assay, suggesting that β-arrestin-2 plays an important role in agonist-dependent internalization of human MC4R (Figures 2E and 2F). Expression levels in EE compartments were comparable upon β-arrestin-1 or -2 knockdown (Figure 2G). Using a NanoBiT protein:protein interaction assay, we found that obesity-associated mutants that did not affect cell surface expression in the basal state frequently impaired the interaction between MC4R and β-arrestin-1, β-arrestin-2, or more frequently both β-arrestins (Figures 2H and 2I). MC4R mutants with decreased PM localization exhibited reduced β-arrestin-1 and -2 recruitment (Figures S4A and S4B). Together, these results demonstrate that recruitment of β-arrestin-2 to MC4R is a key mechanism that governs its internalization and signaling, and one which can be impacted by human obesity-associated and obesity-protecting mutations.

### Regulation of MC4R-mediated ERK1/2 phosphorylation by β-arrestin and Gα_s_-coupled pathways

**Figure 3:**
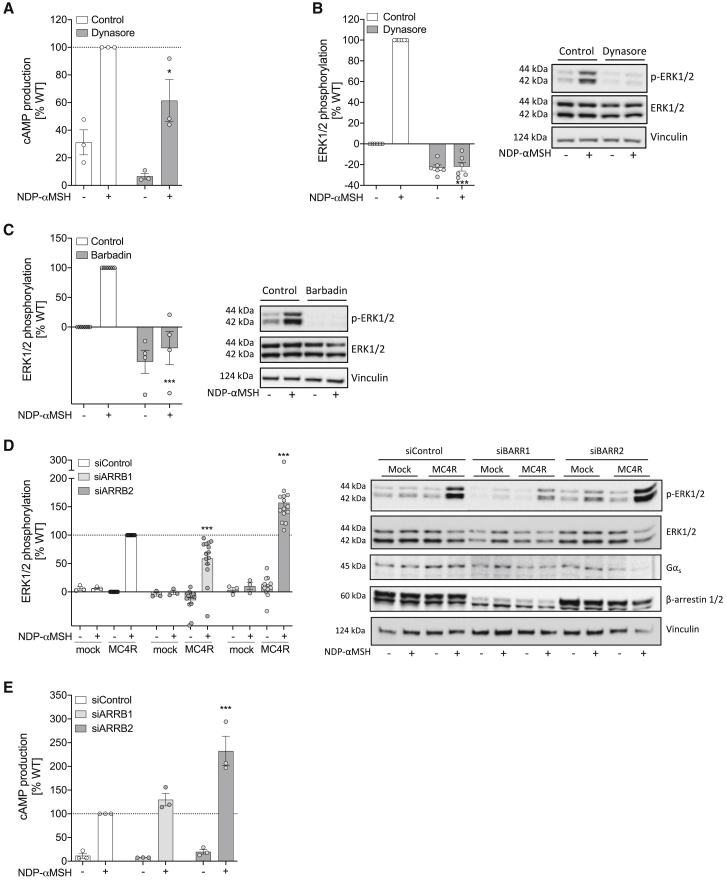
MC4R-derived MAPK activation is dependent on receptor internalization and modulated by β-arrestins (A and B) HEK293 cells transiently expressing MC4R WT were used to assess the effect of dynasore on maximal efficacy of NDP-αMSH in cAMP production (A) and ERK1/2 phosphorylation (B). (C and D) NDP-αMSH-dependent ERK1/2 phosphorylation in HEK293 cells treated with the β-arrestin/AP2 inhibitor barbadin (C) and siControl, siARRB1, or siARRB2 (D). (E) Effect of siControl, siARRB1, or siARRB2 on maximal efficacy of NDP-αMSH-induced cAMP production. Representative immunoblots shown were probed for total ERK1/2, phosphorylated ERK1/2 (p-ERK1/2), Gα_s_, β-arrestin-1/2, and vinculin as an additional loading control. Data are expressed as percentage of maximal control response (% WT) and plotted as mean ± standard error from 3–14 independent experiments. Statistical significance was determined by two-way ANOVA and Dunnet’s multiple comparison test (*p < 0.05, ^∗∗∗^p < 0.001, compared to NDP-αMSH-stimulated MC4R WT [control]). See also Figure S4.

In addition to receptor internalization, β-arrestins desensitize GPCR signaling, hindering the physical interaction between G-protein and receptor while simultaneously initiating β-arrestin-dependent activation of the MAPK pathway, leading to phosphorylation of ERK1/2. For MC4R, we found that inhibition of dynamin-dependent internalization using the GTPase inhibitor dynasore markedly reduced agonist-induced cAMP generation (Figure 3A) and completely abolished ERK1/2 phosphorylation (Figure 3B). Using the selective β-arrestin/β2-adaptin interaction inhibitor barbadin, we showed that β-arrestin-dependent internalization plays an important role in ERK1/2 phosphorylation (Figure 3C). Acute knockdown of *ARRB1* (encoding β-arrestin-1) reduced ERK1/2 phosphorylation, whereas, paradoxically, knockdown of *ARRB2* (encoding β-arrestin-2) increased ERK1/2 phosphorylation (Figure 3D). Opposing effects of β-arrestin-1 versus -2 have been described previously for other GPCRs (Shintani et al., 2018). Cross-talk between G-protein-dependent and G-protein-independent signaling has been demonstrated for a subset of (but not all) GPCRs (Grundmann et al., 2018; Luttrell et al., 2018). We found that acute knockdown of β-arrestin-2 significantly increased ligand-induced cAMP production by MC4R (Figure 3E).

**Figure 4:**
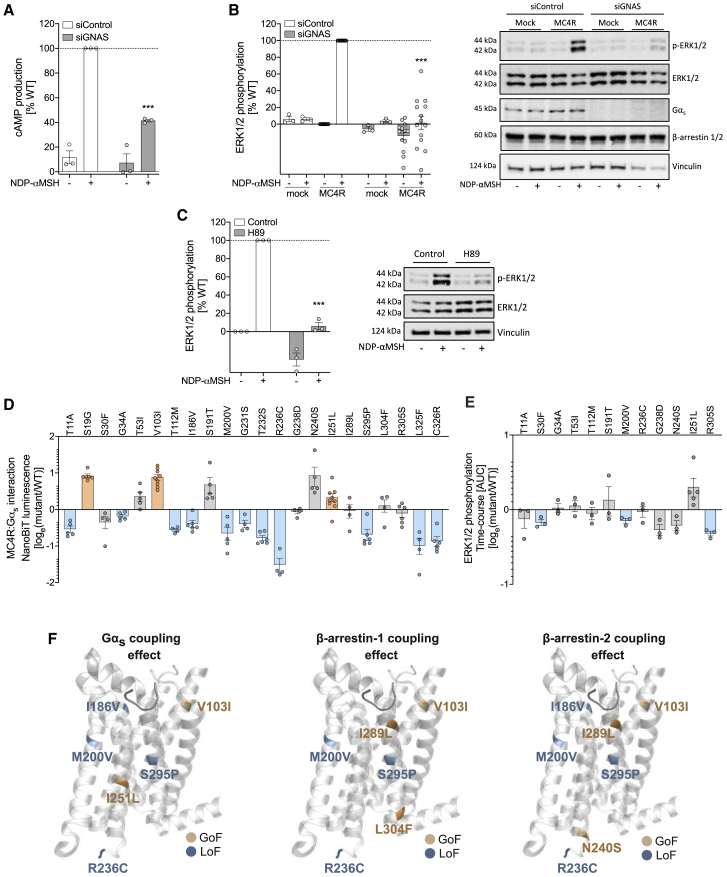
Gα_s_ signaling is essential for MC4R-dependent ERK1/2 phosphorylation, and obesity-associated mutants impair the interaction between Gα_s_ and MC4R (A and B) HEK293 cells transiently expressing WT MC4R were used to assess the effect of siControl or Gα_s_ (siGNAS) on maximal efficacy of NDP-αMSH in cAMP production (A) and ERK1/2 phosphorylation (B). (C) NDP-αMSH-dependent ERK1/2 phosphorylation in HEK293 cells treated with H89 (protein kinase A inhibitor). (D) Agonist-dependent Gα_s_:MC4R interaction for mutants that do not impair PM expression. (E) NDP-αMSH-mediated ERK1/2 phosphorylation for MC4R mutants. (A–C) Data are expressed as percentage of maximal control response (% WT) and plotted as mean ± standard error from 3–14 independent experiments. Statistical significance was determined by two-way ANOVA and Dunnet’s multiple comparison test (^∗∗∗^p < 0.001, compared to NDP-αMSH-stimulated MC4R WT (control)). Representative immunoblots shown were probed for total ERK1/2, phosphorylated ERK1/2 (p-ERK1/2), β-arrestin-1/2, Gα_s_, and vinculin as an additional loading control. (D and E) Data are expressed as log_e_ (mutant/WT) and plotted as mean ± standard error from 3–9 independent experiments. (F) GoF and LoF MC4R mutants affecting coupling to Gα_s_, β-arrestin-1, and β-arrestin-2 are shown mapped into a cartoon representation of the crystallized MC4R receptor structure. The crystallized ligand SHU9119 is shown in dark gray (PDB: 6W25) (Yu et al., 2020). In (D)–(F), mutants were classified as GoF (orange), LoF (blue), or WT-like (gray) based on statistically significant differences between WT and mutant (unpaired t test with Welch’s correction; p < 0.05). See also Figures S4–S6.

We next tested whether Gα_s_ coupling to MC4R is required for agonist-induced ERK1/2 phosphorylation. siRNA-mediated knockdown of *GNAS* (encoding Gα_s_) reduced cAMP production (Figure 4A) and completely inhibited ERK1/2 phosphorylation (Figure 4B). Furthermore, pharmacological inhibition of protein kinase A (PKA), which is activated by increased intracellular cAMP levels, significantly decreased phosphorylation of ERK1/2 (Figure 4C), indicating that Gα_s_ coupling is critical for MC4R-mediated ERK1/2 phosphorylation. Cell lines lacking β-arrestin-1/2 (Figure S4C) and Gα_s_ (Figure S4D) gave variable results, presumably due to compensatory mechanisms observed in these cell lines (Luttrell et al., 2018). Thus, activation of ERK1/2 downstream of MC4R is modulated by both β-arrestin and Gα_s_-coupled pathways. These findings are important, as ERK1/2 activation is often used as a readout in drug discovery studies.

### MC4R mutations impair Gα_s_ coupling, but reduced cAMP accumulation is only detected for a subset

We next used an enzyme complementation assay to study 22 mutants with normal PM expression. We observed a significant reduction in Gα_s_ coupling for 11 mutants (Figure 4D); interestingly, 8 of these were previously described as WT-like in a cAMP accumulation assay (Collet et al., 2017). A likely explanation is that secondary messenger assays rely on signal amplification, so mutants that cause partial loss-of-function (LoF) in G protein coupling can lead to maximal responses that cannot be distinguished from WT receptors. In contrast, in assays based on signal quantification directly related to receptor occupancy, such as β-arrestin recruitment or Gα_s_ coupling, partial LoF mutations give significantly lower maximal responses than WT mutations. In line with this idea, only 2 out of 20 mutants showed significantly decreased maximal signaling in agonist-induced cAMP production (pGloSensor-20F) assays (Figure S4E). Most MC4R mutants that exhibit decreased PM localization show LoF in Gα_s_ coupling and cAMP production (Figures S4F and S4G; Table S1). Similarly, most mutants did not affect MAPK activation, as evidenced by agonist-induced phosphorylation of ERK1/2 (Figure 4E; Figures S4H, S5, and S6). Only MC4R mutations that markedly affected receptor number at the cell surface and Gα_s_ coupling showed significantly reduced ERK1/2 phosphorylation (Figures S4, S5, and S6); some of these also affected β-arrestin recruitment (Table S1). Our findings demonstrate that mutants in MC4R characterized as WT-like in cAMP production and/or ERK1/2 phosphorylation assays can nonetheless impair coupling to Gα_s_ and β-arrestin causing LoF. We found that carriers of these variants displayed the classical features of MC4R deficiency, including severe early-onset obesity, accelerated linear growth, early hyperinsulinemia (Martinelli et al., 2011), and low systolic blood pressure (Greenfield et al., 2009; Table S2), demonstrating that disruption of these mechanisms is sufficient to reduce MC4R function to a level that is physiologically significant.

### Mapping mutants on the 3D structure of MC4R identifies regions involved in differential signaling

The determination of the 3D structure of MC4R in early 2020 represents a major milestone in melanocortin biology (Yu et al., 2020). To understand the structural determinants of MC4R that modulate ligand binding, activation, and signaling, we mapped variants affecting MC4R signaling onto the MC4R receptor structure in complex with the melanocortin antagonist SHU9119 (PDB: 6W25) (Yu et al., 2020). Interestingly, MC4R mutants that impair PM expression, likely due to protein misfolding (Lubrano-Berthelier et al., 2006), tend to be enriched in TM-1 and TM-4 of the receptor (Figure S7A). In contrast, mutants that do not affect expression mostly map either in the N-terminal segment, where they might affect ligand binding, or in receptor segments classically related to receptor activation and coupling to signaling transducers, such as TM-5, the C terminus, and particularly TM-6 and TM-7 (Figure S7B).

We examined the structural determinants of receptor coupling to different effectors. We found that some mutants that map near the ligand binding site, in the central receptor core and in sites that interact with intracellular effectors, exhibit consistent effects on receptor coupling to all of the analyzed intracellular transducers (Figure 4F; V103I, M200V, R236C, and S295P). Some mutations that map to the intracellular side of the receptor seem to be coupling-partner specific, which could be important for the specificity of binding of the two β-arrestins (Figure 4F; L304F and N240S). Interestingly, we also observe mutants near the ligand binding site that could affect which coupling partner is recruited. In the case of I186V and I289L, ligand contacts with any of these residues, or with both, may differently impact the recruitment of Gα_s_, β-arrestin-1 ,or β-arrestin-2. It is likely that specific MC4R mutations stabilize distinct conformational ensembles that may involve conserved residues that determine different receptor activation and coupling states (i.e., microswitches; Smith et al., 2018; Wootten et al., 2018).

### MC4R mutations disrupt receptor homodimerization

**Figure 5:**
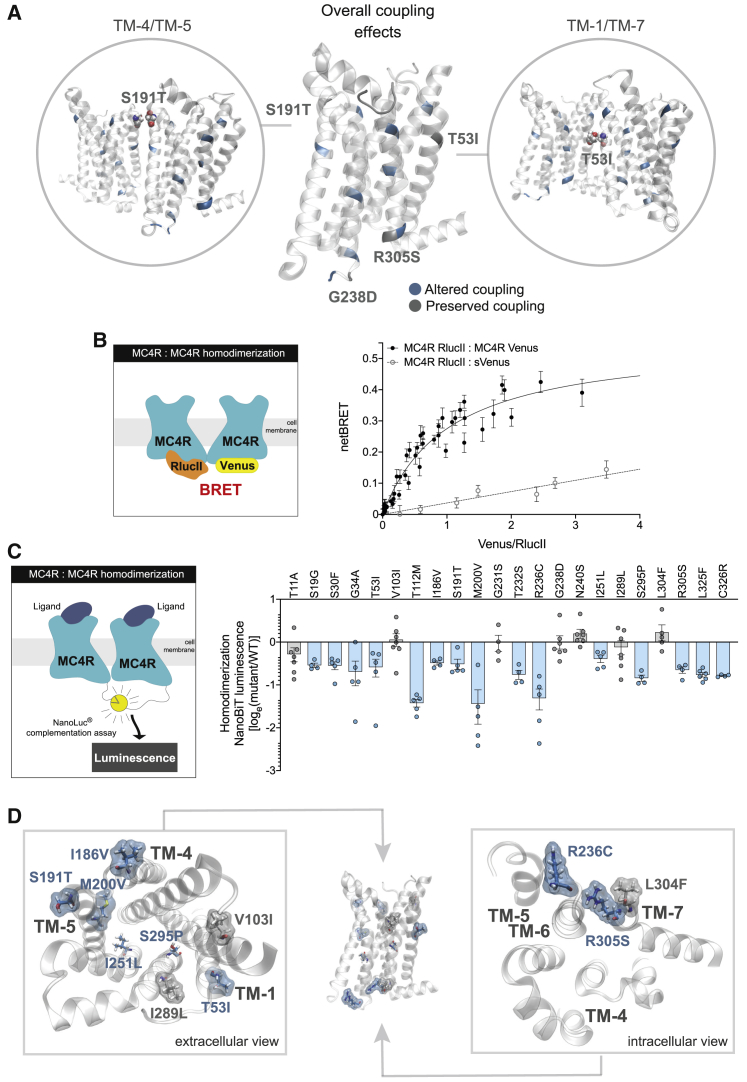
Structural mapping of MC4R mutants suggests impaired dimerization (A) Receptor mutants affecting at least one coupling partner (blue) and those that do not affect coupling (gray). Two of the latter map to one of the proposed MC4R homodimeric interfaces, being within 4Å of the opposite MC4R partner (shown in gray in a van der Waals representation). S191T maps to a TM-4/TM-5 interface (left inset); T53I maps to a TM-1 /TM-7 interface (right inset). (B) BRET saturation curve from HEK293SL cells co-transfected with a constant amount of MC4R RlucII donor construct and increasing amounts of the MC4R Venus acceptor construct. Soluble (s) Venus acceptor construct was used as a negative control. (C) MC4R WT:MC4R mutant receptor dimerization as measured in NanoBiT protein:protein interaction assay (see STAR Methods). Data are expressed as log_e_ (mutant/WT) and plotted as mean ± standard error from 4–7 independent experiments. Mutants were classified as LoF (blue) or WT-like (gray) based on statistically significant differences between WT and mutant (unpaired t test with Welch’s correction; p < 0.05). (D) Structural mapping of variants affecting dimerization (blue sticks) and variants with preserved dimerization (gray sticks). Membrane-facing residues are highlighted in a surface representation. The left panel shows details from a top (extracellular) view of the receptor, whereas the right panel shows a bottom (intracellular) view. See also Figure S4.

We then focused on mutants that do not have measurable effects on PM expression (Figures 1C and 5A). The location of G238D in ICL3 of the receptor suggests an effect on membrane trafficking, which was supported by our experimental findings that demonstrate impaired internalization of this mutant (Figures 1D and 1E; Figures S2 and S3).The effects of three remaining mutations (R350S, S191T, and T53I), however, remain more difficult to predict, which is especially true in the case of S191T and T53I that face the PM and as such do not constitute the ligand or the signal transducer binding sites directly. However, their positions map into two previously proposed MC4R homodimer interfaces (Heyder et al., 2019). When we model these homodimers (see STAR Methods), we observe that S191T is found at a TM-4/TM-5 receptor interface (Figure 5A, left inset), whereas T53I maps to a TM-1/TM-7 interface (Figure 5A, right inset). These observations suggest that these mutants may effect MC4R homodimerization.

To test whether MC4R can homodimerize, we first used a BRET-based assay. We observed a saturable curve (BRET_50_, 1.037; BRET_max_, 0.56) in cells expressing a constant amount of donor construct (MC4R RlucII) and increasing amounts of acceptor construct (MC4R Venus), indicating a specific and saturable MC4R:MC4R interaction (Figure 5B). The selectivity of the observed signal was further supported by the fact that co-expression of MC4R RlucII with a soluble acceptor (sVenus) led to lower BRET signals that progressed linearly over the same range of acceptor/donor ratios (Figure 5B). In addition, using the NanoBiT protein:protein interaction assay, we detected a robust interaction between two differentially tagged protomers of MC4R, establishing that MC4R can indeed homodimerize (Figure 5C). We tested 22 MC4R mutants with normal PM expression for their ability to dimerize with WT MC4R and found that multiple mutations, including those affecting the putative dimerization interfaces (T53I and S191T), decreased dimerization with WT MC4R (Figure 5C). A subset of mutants that decrease receptor dimerization appear to be surface exposed and facing the proposed dimerization interfaces (I186V and M200V; Figure 5D). For other variants facing the receptor core (I251L and S295P; Figure 5D), effects on dimerization may not depend on direct contacts between protomers but instead on allosteric effects on receptor conformation. In the intracellular part of the receptor, exposed variants affecting dimerization (R305S and R236C; Figure 5D) do not directly face the classical interfaces, suggesting effects on helix packing or, alternatively, additional receptor-receptor contact sites.

## Discussion

**Figure 6:**
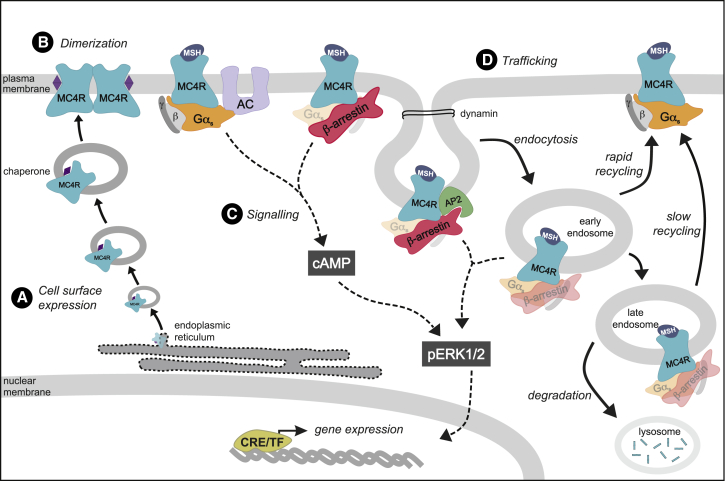
Obesity-associated and obesity-protecting mutations in MC4R affect multiple molecular mechanisms (A) GoF mutations increase, whereas LoF MC4R mutations decrease receptor numbers at the cell surface. Molecular chaperones (heat shock proteins and MRAP2), chemical chaperones (e.g., 4-phenylbutyrate), and pharmacological chaperones (antagonists and agonists) have the potential to stabilize and target intracellularly retained MC4R mutants to the PM (chaperones represented as purple diamond). (B) MC4Rs homodimerize, and obesity-associated mutations can disrupt this process. (C) Production of the second messenger cAMP is dependent on Gα_s_ protein and modulated by β-arrestins; MC4R mutations can differentially affect coupling to these signaling transducers. G protein-induced cAMP production is essential for MAPK activation (ERK1/2), which leads to changes in gene expression by activation of cAMP response element-binding protein (CREB-TF). (D) Agonist-dependent endocytosis of MC4R is mainly driven by β-arrestins and is critical for ERK1/2 activation. Several mutations in MC4R negatively affect the sequestration of receptors from the PM, the translocation to EEs, or both. GoF mutations cycle more avidly by rapid recycling pathways (EE to PM), rather than being directed to late compartments (slow recycling) or degradative pathways (lysosomes) and as such are more available at the surface for subsequent rounds of agonist-induced signaling. Semi-transparent (faded) symbols for G protein subunits and β-arrestins indicate potential involvement of these intracellular effectors in megaplexes (Thomsen et al., 2016), although this has not been formally evaluated in this study.

MC4R plays a pivotal role in the regulation of body weight, as demonstrated in animal models and in humans, in which variants that cause a LoF by reducing cAMP production are associated with obesity (Farooqi et al., 2003; Hinney et al., 2006; Stutzmann et al., 2008). Moreover, gain-of-function MC4R variants are associated with protection from obesity (Lotta et al., 2019). Here, in a series of studies using naturally occurring human *MC4R* variants, we provide compelling evidence that human MC4R homodimerizes, undergoes agonist-promoted β-arrestin-mediated endocytosis, and that both G protein-dependent and G protein-independent signaling contribute to ERK1/2 phosphorylation (Figure 6). In keeping with previous studies, we showed that missense mutations in MC4R reduce PM expression (Lubrano-Berthelier et al., 2006). In addition, we showed that independently of effects on PM expression, MC4R mutations can disrupt endocytosis and intracellular trafficking. Carriers of variants that disrupt one or more of these mechanisms share the clinical features of MC4R deficiency reported by us and others (Farooqi et al., 2003; Lubrano-Berthelier et al., 2006; Table S2), demonstrating that these mechanisms act as control points to alter melanocortin tone to a level that is physiologically relevant in humans. Due to the rarity of these variants, transgenic mouse models of specific human MC4R mutations will be needed to further dissect genotype-phenotype correlations.

Our work expands current understanding of the mechanisms involved in the trafficking of and signaling by MC4R. The endocytosis of MC4R seems to be primarily driven by β-arrestin-2, as seen previously for the β2-adrenergic receptor (β2AR) (Kohout et al., 2001), whereas for other GPCRs, e.g., the angiotensin II type 1A receptor (AT1AR), both β-arrestins contribute equally to endocytosis. We find that β-arrestin-1 and -2 differentially effect ERK1/2 phosphorylation and cAMP signaling downstream of MC4R, as seen for some but not all other GPCRs (Kuo et al., 2006; Paing et al., 2002). Further studies of conditional mouse models will be needed to dissect the physiological contribution of differences in trafficking and ERK1/2 phosphorylation mediated by the β-arrestins.

The findings presented here have diagnostic and clinical implications. We found that all 19 rare *MC4R* variants previously thought to have no/limited effects on cAMP production (Collet et al., 2017) do in fact impair PM and/or EE localization, interaction with β-arrestins or Gα_s_ protein, and receptor internalization and/or homodimerization. These mutations therefore cause a LoF in cells and can be classified as pathogenic (Richards et al., 2015). One example is F202L, a variant that is more prevalent in people of African ancestry (MAF, 0.9%) (Logan et al., 2015) and was found in 6 unrelated people of African ancestry in the Genetics of Obesity Study (GOOS) cohort (n = 7,447 screened). Our findings establish a role for this variant in weight gain in people of African ancestry (De Rosa et al., 2019; Logan et al., 2015) with implications for carriers who may benefit from treatments that are effective in MC4R deficiency. People with heterozygous MC4R deficiency can respond to treatment with the GLP-1 receptor agonist liraglutide (Iepsen et al., 2018) and with Roux-en-Y-bypass surgery (Hatoum et al., 2012), but those with homozygous MC4R mutations may not respond to bypass surgery, although they may to GLP1 receptor agonism (Iepsen et al., 2020).

To date, several therapeutic strategies for targeting MC4R signaling have been explored. First-generation melanocortin receptor agonists were effective for weight loss (Sharma et al., 2019) but were associated with increased blood pressure, as predicted by studies in *MC4R*-deficient mice and humans (Greenfield et al., 2009; Tallam et al., 2005). Setmelanotide, a second-generation small-molecule MC4R agonist currently in clinical trials, causes significant weight loss in complete POMC deficiency (Kühnen et al., 2016) and other genetic obesity syndromes characterized by a loss of melanocortin tone (Clément et al., 2018, 2020; Collet et al., 2017; Haws et al., 2020). Interestingly, setmelanotide does not increase blood pressure. Whether the effects of MC4R agonism on blood pressure and other phenotypes might be explained by some of the cellular mechanisms explored in this study remains to be determined.

A number of studies have shown that pharmacological chaperones can restore PM expression by stabilizing and rescuing misfolded receptors trapped within the cell’s quality-control system (endoplasmic reticulum and Golgi apparatus) (Granell et al., 2010; René et al., 2010). Moreover, endogenous molecular chaperones critical for maintaining protein homeostasis, such as the heat shock proteins (Hsp70/Hsp90 chaperone system), can modulate trafficking of WT MC4R and rescue the cell surface expression and function of inherited mutations in MC4R (Meimaridou et al., 2011). Additionally, the melanocortin accessory protein MRAP2 has been shown to modulate trafficking and function of MC4R (Sebag et al., 2013) to a level that is physiologically significant, as transgenic mice with whole-body deletion and brain-specific deletion of *Mrap2* develop severe obesity at a young age (Asai et al., 2013). Harnessing endogenous chaperones and/or developing pharmacological chaperones represent attractive strategies to enhance melanocortin signaling for clinical benefit.

The development of ligands that preferentially engage G protein-dependent or β-arrestin-dependent signaling, a concept known as biased signaling or functional selectivity, is an area of much therapeutic interest (Rajagopal et al., 2011; Smith et al., 2018). Our structural mapping of receptor mutants revealed ligand-accessible residues with a differential impact on receptor coupling. Designing ligands that contact these positions to mimic the effect of specific MC4R mutants may represent an effective strategy. We performed preliminary studies of signaling by endogenous POMC-derived peptides (α-, β-, and γ-MSH) and commercially available synthetic peptides in stably transfected cells expressing human WT MC4R with cAMP GloSensor or a β-arrestin reporter (Figure S8A). We found that all γ-MSH-related and the majority of α-MSH-related peptides preferentially activated β-arrestin-2 recruitment (Figure S8B). Further studies of these compounds using assays with the same kinetics and that are not confounded by differential signal amplification (e.g., BRET Gα_s_ and BRET β-arrestin) will be necessary to precisely calculate functional selectivity (Gillis et al., 2020). Whether or not biased agonists offer therapeutic benefits beyond those that might be achieved with balanced agonists remains to be determined for MC4R.

Based on structural mapping of obesity-associated mutants, we predicted that human MC4R can form homodimers; this assertion was validated in cells using two independent experimental approaches. One previous study showed that a *MC4R* variant (D90N) exerted a dominant-negative effect on WT MC4R and led to the suggestion that MC4R may exist as dimers or oligomers (Biebermann et al., 2003). In these studies, dimerization between differentially tagged MC4R protomers was assessed by sandwich ELISA and fluorescence resonance energy transfer (FRET). However, colocalization techniques may not provide sufficient subcellular resolution to establish close proximity; coimmunoprecipitation of differentially tagged receptors can occur even if the receptors are too far apart to allosterically modulate one another in a complex (Ferré et al., 2014). Here, we established MC4R homodimerization by using saturation BRET, allowing us to determine the specificity of the interaction, and used a second method, the NanoBiT protein:protein interaction assay, to detect homodimerization at lower expression levels. Both methods are validated for GPCR oligomer detection (Guo et al., 2017). In the present study, we delineate specific regions involved in dimerization and show that several human obesity-associated mutations disrupt this process when studied in cells. Some MC4R mutations (e.g., T53I) directly impact the dimerization interface, as seen for inherited mutations in the thromboxane A2 receptor (Capra et al., 2017), whereas other mutations impact dimerization in ways that are incompletely understood. Further studies will be needed to dissect how these mutations affect the impact of one protomer on signaling by the other, internalization of ligand-receptor complexes, and the kinetics or affinity of binding to β-arrestin, as seen for other GPCRs (Ferré et al., 2014). MC4R dynamics upon binding of ligands by using solution-state nuclear magnetic resonance spectroscopy and molecular dynamic simulations might provide more detailed insights into the molecular basis of preferential activation of G protein-dependent versus G protein-independent signaling by MC4R. We recognize that the structural information currently available for MC4R, which represents an inactive conformation of the receptor, in part limits our structural interpretation of variants. Additional insights may emerge from structures of the receptor in complex with different coupling partners and of the active receptor solved in its homodimeric organization in the future.

Our findings suggest that targeting MC4R homodimers might present an attractive therapeutic strategy, for example, through the use of bitopic ligands that bind to one MC4R protomer allosterically, modulating ligand binding to the orthosteric site of the second MC4R protomer (Lane et al., 2014). Allosteric modulation of GPCRs at sites that are distinct from binding sites for endogenous ligands can provide higher selectivity at the target and can alter the kinetics of binding affinity and receptor activation for therapeutic benefit. Interestingly, several of the allosteric modulators currently in clinical trials are combination therapies with orthosteric ligands for the treatment of CNS disorders (Nickols and Conn, 2014).

In summary, by dissecting mechanisms that control MC4R signaling by using naturally occurring human mutations, we link mechanisms to phenotype and thereby expand our understanding of how to most effectively target a key GPCR involved in weight regulation for the treatment of obesity and its complications.

## STAR★Methods

### Key resources table

**Table:** 

REAGENT or RESOURCE	SOURCE	IDENTIFIER
**Antibodies**		

Rabbit anti-p44/42 MAPK (Erk1/2) (137F5)	Cell Signaling Technology	Cat#4695; RRID: AB_390779
Rabbit anti-Phospho-p44/42 MAPK (Erk1/2) (Thr202/Tyr204)	Cell Signaling Technology	Cat#9101; RRID: AB_331646
Goat anti-GNAS	Thermo	Cat#PA5-19315; RRID: AB_10983686
Rabbit-Anti-Vinculin [EPR8185]	Abcam	Cat#ab129002; RRID: AB_11144129
Goat anti-rabbit IgG-HRP	Dako	Cat#P0448; RRID: AB_2617138
Donkey anti-Goat IgG-HRP	Santa cruz	Cat#sc-2056 RRID: AB_631730
Anti-HA-HRP (3F10)	Sigma-Aldrich	Cat#12013819001; RRID: AB_390917
Mouse monoclonal anti-FLAG (M2) antibody	Sigma-Aldrich	Cat#F1804; RRID: AB_262044
Goat anti-mouse IgG-HRP	Dako	Cat#P0447; RRID: AB_2617137

**Bacterial and virus strains**		

XL10-Gold	Agilent	Cat# 200315

**Chemicals, peptides, and recombinant proteins**		

Dulbecco’s modified eagle medium (high glucose DMEM)	GIBCO	Cat#41965
Fetal Bovine Serum (FBS)	GIBCO	Cat#10270
GlutaMAX™	GIBCO	Cat#35050
Lipofectamine 2000	Invitrogen	Cat#11668
Lipofectamine RNAiMAX	Invitrogen	Cat#13778150
25 kDa linear polyethylenimine (PEI)	Alfa Aesar	Cat#43896
Coelenterazine 400a	NanoLight Technology	Cat#340
Prolume Purple	NanoLight Technology	Cat#369
NDP-αMSH	Bachem	Cat#H-1100
Dynasore	Tocris	Cat#2897
Barbadin	Aobious	Cat#AOB 37364
H89	Sigma-Aldrich	Cat#B1427

**Critical commercial assays**		

QuikChange II XL kit	Agilent Technologies	Cat#200516
GloSensor™ cAMP biosensor - pGloSensor™ 20F plasmid	Promega®	Cat#E1171
GloSensor™ cAMP Reagent	Promega®	Cat#E1290
NanoBiT protein:protein interaction assay	Promega®	Cat#M2014
Nano-Glo® Live Cell Assay System	Promega®	Cat#M2013

**Experimental models: cell lines**		

HEK293	ATCC	CRL-1573; RRID: CVCL_0045
COS-7	ATCC	CRL-1651; RRID: CVCL_0224
HEK293SL	Lotta et al., 2019	n/a
HEK293AI parental	O’Hayre et al., 2017	n/a
HEK293AI ARRB1/2 KO	O’Hayre et al., 2017	n/a
HEK293AI GNAS KO	O’Hayre et al., 2017	n/a
HEK293 MC4R: pGloSensor-20F Stable cell line	This paper	n/a
HEK293 MC4R:β-arrrestin-2 Stable cell line	This paper	n/a

**Oligonucleotides**		

siGENOME Human GNAS siRNA SMARTPool	Dharmacon	Cat#M-010825-02-0005
siGENOME Human ARRB1 siRNA SMARTPool	Dharmacon	Cat#M-011971-01-0005
siGENOME Human ARRB2 siRNA SMARTPool	Dharmacon	Cat#M-007292-00-0005
siGENOME siRNA Non-Targeting Control Pool 1	Dharmacon	Cat#D-001206-13-05

**Recombinant DNA**		

Human N-FLAG-MC4R-WT in pCDNA3.1(+) vector	Luttrell et al., 2018	n/a
Human N-FLAG-MC4R variants in pCDNA3.1(+) vector	This paper	n/a
Human N-3x(HA)-MC4R-C-RlucII WT in pCDNA3.1(+) vector	Kindly provided by Prof. Michel Bouvier (University of Montreal)	n/a
Human N-3x(HA)-MC4R-C-RlucII variants in pCDNA3.1(+) vector	This paper	n/a
Human MC4R-WT-C-LgBiT in pBiT1.1-C[TK/LgBiT] vector	Lotta et al., 2019	n/a
Human MC4R-WT-C-LgBiT in pBiT2.1-C[TK/SmBiT] vector	This paper	n/a
Human MC4R-C-LgBiT variants in pBiT1.1-C[TK/LgBiT] vector	This paper	n/a
Human N-SmBiT-ARRB1 in pBiT2.1-N[TK/SmBiT] vector	This paper	n/a
Human N-SmBiT-ARRB2 in pBiT2.1-N[TK/SmBiT] vector	Lotta et al., 2019	n/a
Human Gα_s_-C-SmBiT in pBiT2.1-C[TK/SmBiT] vector	This paper	n/a
Human MC4R-WT-C-LgBiT and Human N-SmBiT-ARRB2 in NanoBiT CMV MCS BiBiT vector	This paper	n/a
rGFP-CAAX in pCDNA3.1(+) vector	Namkung et al., 2016	n/a
rGFP-FYVE in pCDNA3.1(+) vector	Namkung et al., 2016	n/a
drGFP-Rab7 in pCDNA3.1(+) vector	Kindly provided by Prof. Michel Bouvier (University of Montreal)	n/a
Human N-3x(HA)-MC4R-C-venus WT in pCDNA3.1(+) vector	Kindly provided by Prof. Michel Bouvier (University of Montreal)	n/a

**Software and algorithms**		

GraphPad Prism version 8	Graph Pad Software, Inc	https://www.graphpad.com
Geneious version 9	Geneious	https://www.geneious.com
FIJI	Schindelin et al., 2012	https://fiji.sc
VMD 1.9.3 software	Humphrey et al., 1996	http://www.ks.uiuc.edu/Research/vmd/

### Resource availability

#### Lead contact

Further information and requests for resources and reagents should be directed to and will be fulfilled by Sadaf Farooqi (isf20@cam.ac.uk).

#### Materials availability

Plasmids and cell lines generated for this study are available upon request with a completed materials transfer agreement (MTA).

#### Data and code availability

This study did not generate any unique datasets or code.

### Experimental model and subject details

#### Studies in humans

All genetic and clinical studies were approved by the Multi-Regional Ethics Committee and the Cambridge Local Research Ethics Committee (MREC 97/21 and REC number 03/103 respectively). Each subject (or their parent for those under 16 years) provided written informed consent; minors provided oral consent. All research was conducted in line with the principles outlined in the Declaration of Helsinki. The Genetics of Obesity Study (GOOS) is a cohort of over 7,000 individuals with severe early-onset obesity; age of obesity onset is less than 10 years (Farooqi et al., 2003). Severe obesity is defined as a body mass index (weight in kilograms divided by the square of the height in meters) standard deviation score greater than 3 (standard deviation scores calculated according to the United Kingdom reference population). Plasma insulin and glucose were measured in the fasting state using standard assays.

### Method details

#### *In vitro* mutagenesis of MC4R

MC4R cDNA constructs containing an N-terminal FLAG tag in pcDNA3.1(+), triple tandem N-terminal HA tags and a C-terminal RlucII tag in pcDNA3.1(+) or a C-terminal LgBiT tag in pBiT1.1-C[TK/LgBiT] vector were used throughout the study. Site-directed mutagenesis was performed using QuikChange II XL kit (Agilent Technologies, 200516) according to the manufacturer’s protocols. All constructs were verified by Sanger sequencing before use.

#### Cell culture and transfection

In order to characterize the functional consequences of MC4R mutants we performed *in vitro* assays in transiently transfected HEK293, HEK293SL (Luttrell et al., 2018), HEK293AI cells (O’Hayre et al., 2017) and COS7 cells maintained in Dulbecco’s modified eagle medium (high glucose DMEM, GIBCO, 41965) supplemented with 10% fetal bovine serum (GIBCO, 10270), 1% GlutaMAX™ (100X) (GIBCO, 35050), and 100 units/mL penicillin and 100 μg/mL streptomycin (Sigma-Aldrich, P0781). Cells were incubated at 37°C in humidified air containing 5% CO_2_ and transfections were performed using Lipofectamine 2000 (GIBCO, 11668) in serum-free Opti-MEM I medium (GIBCO, 31985) or 25 kDa linear polyethylenimine (PEI; Alfa Aesar), according to the manufacturer’s protocols, unless stated otherwise.

#### Enhanced bystander bioluminescence resonance energy transfer (ebBRET) to measure MC4R internalisation and cell surface expression

Transient transfections were performed in a HEK293SL cell suspension at a density of 3 × 10^5^ cells/mL using 25 kDa linear polyethylenimine (PEI; Alfa Aesar) as transfecting agent, at a ratio of 3:1 PEI/DNA. For experiments with rGFP-CAAX (PM sensor), 0.2 μg of receptor plasmid (3xHA-MC4R-RlucII) was combined with 0.1 μg of rGFP-CAAX. For rGFP-FYVE (EE sensor), 0.1 μg receptor plasmid was combined with 0.3 μg of rGFP-FYVE, and for the drGFP-Rab7 (LE sensor), 0.025 μg receptor plasmid was combined with 0.3 μg of drGFP-Rab7. Plasmid DNAs were completed to 1 μg with salmon sperm DNA (Invitrogen) and the final mixture was used to transfect 3.6 × 10^5^ cells. Transfected cells were distributed in white 96-well microplates (100 μL/well; Greiner) for BRET assays. Cells were maintained in culture for 48 hours before performing assays.

Culture medium from microplates was removed; cells were washed with Dulbecco’s phosphate-buffered solution (D-PBS; GIBCO) to remove phenol red and replaced by Tyrode’s buffer (NaCl 137 mM, KCl 0.9 M, MgCl_2_ 1 mM, NaHCO_3_ 11.9 mM, NaH_2_PO_4_ 3.6 mM, HEPES 25 mM, glucose 5.5 mM, CaCl_2_ 1 mM, pH 7.4). After addition of NDP-α-MSH or vehicle, cells were incubated at 37°C for 30 min (rGFP-FYVE and drGFP-Rab7 sensors) or 60 min (rGFP-CAAX sensor) and luciferase substrate coelenterazine 400a (2.5 uM; NanoLight Technology) was added 5 (rGFP-FYVE and drGFP-Rab7 sensors) or 15 (rGFP-CAAX sensor) minutes prior ebBRET reading in a FLUOstar Omega microplate reader (BMG Labtech) equipped with acceptor filter (515 ± 30 nm) and donor filter (410 ± 80 nm), or a Mithras LB940 Multimode Microplate Reader (Berthold Technologies), equipped with acceptor filter (515 ± 20 nm) and donor filter (400 ± 70 nm). The ebBRET signal was determined as the ratio of the light emitted by rGFP-CAAX or rGFP-FYVE (energy acceptors) and the light emitted by RlucII-tagged receptors (energy donors). The NDP-α-MSH-promoted ebBRET signal (ΔebBRET) refers to absolute difference of ebBRET recorded from cells treated with agonist and cells treated with vehicle. Internalisation is expressed as percent of the maximal ΔebBRET obtained with wild-type receptor. Values of ebBRET obtained in absence of NDP-α-MSH (vehicle, basal ebBRET) for each RlucII-tagged receptor were used to evaluate the receptor cell surface expression. Values were expressed as percent of basal ebBRET obtained with wild-type MC4R. For quantifying agonist-promoted internalisation, data was normalized to basal ebBRET as measured using the rGFP-CAAX sensor, to control for basal expression of receptors at the PM. Results are from 3 independent experiments (PM), 3-6 independent experiments (EE) and 4 independent experiments (LE). For kinetics experiments, cells were transfected as described above for the PM and EE sensors. Forty-eight hours post transfection, cells were washed once with Tyrode’s buffer, and the luciferase substrate Prolume Purple (NanoLight Technology) was added at a final concentration of 2.5 μM. Cells were treated with 1 μM NDP-α-MSH or vehicle and BRET was measured continuously for ∼40 minutes on a Mithras LB940 Multimode Microplate Reader (Berthold Technologies), equipped with acceptor filter (515 ± 20 nm) and donor filter (400 ± 70 nm). Data are expressed as ΔebBRET (ebBRET_stimulated_-ebBRET_vehicle_) and represent mean ± SEM from 3 independent experiments.

For experiments interrogating the role of β-arrestins in endocytosis, β-arrestin-1/2 double knock-out (ARRB1/2 KO) HEK293SL cells and parental HEK293SL controls were transfected with PEI as above using 0.05 μg of receptor plasmid and 0.4 μg rGFP-CAAX or 0.3 μg rGFP-FYVE, with or without 0.2 μg β-arrestin-2, completed to 1 μg total with empty pcDNA3.1(+) vector. BRET assays were done 48h later as described above, with an agonist stimulation time of 60 min. Coelenterazine 400a (2.5 μM; NanoLight Technology) was added 5 minutes prior to ebBRET reading on a TriStar^2^ machine with acceptor filter (515 ± 25 nm) and donor filter (400 ± 20 nm). Results are from 3 independent experiments.

#### BRET to measure dimerization

Dimerization of MC4R was quantified using BRET1 in titration configuration. Briefly, HEK293SL cells seeded in 96-well plates were transfected with a constant dose of MC4R RlucII plasmid (0.5 ng/well) and increasing doses of MC4R Venus plasmids, or soluble (s) Venus as negative control. All conditions were topped up with empty vector (pcDNA3.1 (+)) to a total of 100 ng plasmid/well. Twenty-four hours post transfection, cells were washed once with Thyrode’s buffer and total Venus fluorescence was measured in a Spark 10M Microplate reader (Tecan) using monochromators (excitation 485 ± 20 nm, emission 535 ± 20 nm). BRET was quantified 10 minutes after the addition of coelenterazine H (NanoLight Technology, 2.5 μM). netBRET was calculated as [(absorbance at 533 ± 25 nm/absorbance at 480 ± 40 nm)] – [background (absorbance at 533 ± 25 nm/absorbance at 480 ± 40 nm)], with the background corresponding to the signal in cells expressing the RlucII protomer alone under similar conditions. Data on the x axis represent the ratio between acceptor (Venus) fluorescence and donor (RlucII) luminescence.

#### Measurement of cell surface expression by enzyme-linked immunosorbent assay (ELISA)

Transient transfections for ELISA were done in parallel to experiments with the rGFP-CAAX sensor described above using the same transfection mix with the 3xHA-MC4R-RlucII plasmid. Transfected cells were distributed in poly-D-lysine (GIBCO)-pretreated white 96-well microplates (100 μl/well; Greiner) for ELISA. Cells were maintained in culture for 48 hours before performing assays. Culture medium was removed, and cells were fixed with D-PBS containing 4% paraformaldehyde for 10 min. Cells were washed 3 times in washing solution (0.5% bovine serum albumin (BSA) in D-PBS) and subsequently incubated for 1 hour with monoclonal anti-HA-HRP 3F10 antibody (1:2000 in washing solution; Sigma-Aldrich). After 3 washes with the washing solution, cells were washed 3 times with PBS to remove BSA. HRP activity was detected by incubating cells with *o*-phenylenediamine dihydrochloride (SigmaFast; Sigma-Aldrich). Reaction was stopped by adding HCl (0.6M final concentration). Solutions were transferred in a transparent 96-well microplate (Greiner) and absorbance was read at 492 nM using a FLUOstar Omega microplate reader (BMG Labtech). Results are from 3-4 independent experiments.

#### Radioligand competition binding assay

Ligand-receptor interaction was assessed in a competition radioligand binding assay in HEK293 cells transiently transfected with 20 ng/well cDNA for MC4R variant or pcDNA3.1(+) (negative control mock transfection) using Lipofectamine 2000. Assays were performed on cells seeded in white 96-well plates and cultured in standard conditions one day after transfection. Prior to the assay, cells were gently washed (200 μl/well) and incubated on wet ice with ice-cold binding assay buffer (50 μl/well, 200 mM HEPES, pH 7.4 supplemented with 119 mM NaCl, 4.7 mM KCl, 5 mM MgCl2, 5.5 mM glucose, 1 mg/ml BSA). Standard serial dilutions of unlabelled NDP-α-MSH (1 pM – 1 μM, 5 μl/well) were dispensed first, swiftly followed by adding 50 μl / well radiolabelled tracer, i.e., [^125^I]-NDP-α-MSH (Perkin Elmer, NEX352). Cells were incubated for at least 3h at 4°C, followed by washing with ice-cold binding assay buffer (2 × 200 μl/well). After final aspiration, 100 μl/well scintillation fluid (MicroScint-20, Perkin Elmer, 6013621) was added, plates were sealed with TopSeal-A PLUS (Perkin Elmer, 6050185) and subsequently shaken for 5 min at 1000 rpm. The activity of a radiolabelled tracer bound was quantified after at least 3 hour settle time using TopCount 9012 Microplate Counter (Packard). Results are from 3-5 independent experiments.

#### Protein: protein interaction assay

Coupling between MC4R and β-arrestin-1, β-arrestin-2 or Gα_s_ was monitored using NanoBiT protein:protein interaction assay (Promega®, M2014). MC4R WT and mutants were cloned into pBiT1.1-C [TK/LgBiT] vector; β-arrestin-1 and β-arrestin-2 into pBiT2.1-N [TK/SmBiT] vector; and Gα_s_ into pBiT2.1-C [TK/SmBiT] vector. Assays were performed in HEK293 cells seeded in poly-D-lysine-coated, white 96-well plates (40,000 cells/well) transiently transfected with 50 ng/well of each of the two constructs as specified previously (Lotta et al., 2019). For the negative control, SmBiT-β-arrestin-1, SmBiT-β-arrestin-2, Gα_s_-SmBiT constructs were substituted with NanoBiT negative control vector (HaloTag-SmBiT). Positive control consisted of SmBiT-PRKACA and LgBiT-PRKAR2A vectors and was added to every experiment. Following the transfection, cells were maintained overnight in cell culture medium. The next day, half an hour prior to the assay, culture medium was substituted for 100 μl/well serum-free Opti-MEM I medium (GIBCO, 31985). Nano-luciferase activity was measured at 37°C and 5% CO_2_ using a Spark 10M microplate reader (Tecan). After initial measurement of the background signal, 25 μl/well Nano-Glo® Live Cell Assay System (Promega, N2013) was added and cells were equilibrated while basal luciferase activity was measured for 10 minutes (1-minute intervals). Subsequently, cells were stimulated with 10 μL of 13.5x stock solution of the MC4R agonist NDP-α-MSH (final concentration 1 μM), and chemiluminescent signal was quantified for 45 minutes (30 s intervals). The area under the curve (AUC) was calculated for each MC4R mutant using the average value for the negative control as the baseline and the total peak of each curve. For data normalization, the AUC from the negative control was set as 0 and the AUC from WT MC4R was set as 100%. Results are from 3-9 independent experiments.

#### Time-resolved cAMP assay

Measurement of ligand-induced cAMP generation in HEK293 cells transiently expressing WT MC4R or mutants was performed using GloSensor™ cAMP biosensor (Promega) according to manufacturer’s protocol. Briefly, 40,000 cells were seeded in white 96-well poly-D-lysine-coated plates. After 24 hours, cells were transfected with both 100 ng/well of pGloSensor™-20F cAMP plasmid (Promega, E1171) and 30 ng/well of plasmid encoding either MC4R WT or MC4R mutants (MC4R-FLAG in pcDNA3.1(+)), using Lipofectamine 2000 (GIBCO, 11668). The day after transfection, cell media were replaced by 90 μL of fresh DMEM with 2% v/v GloSensor™ cAMP Reagent (Promega, E1290) and incubated for 120 min at 37°C. When appropriate, 80 μM dynasore, 100 μM barbadin or DMSO (vehicle) was added to the well 30 minutes before luminescence measurement. Firefly luciferase activity was measured at 37°C and 5% CO_2_ using a Spark 10M microplate reader (Tecan). After initial measurement of the baseline signal for 10 min (30 s intervals), cells were stimulated with 10 μL of 10x stock solution of the MC4R agonist NDP-α-MSH (final concentration 1 μM) and real-time chemiluminescent signals were quantified for 45 minutes (30 s intervals). For gene knockdown experiments using siRNA, 20,000 HEK293 cells were transfected with 10 nM siBARR1, siBARR2, siGNAS or siControl (non-targeting siRNA) 24 hours prior to pGloSensor™-20F cAMP and MC4R plasmids transfection, using Lipofectamine RNAiMAX (Invitrogen, 13778150); siRNA (Dharmacon; siGenome; GNAS, M-010825-02-0005; ARRB1, M-011971-01-0005; ARRB2, M-007292-00-0005 and siControl, D-001206-13-05). In each experiment, a negative control using mock transfected cells (empty pcDNA3.1(+) plasmid) was assayed. The area under the curve (AUC) was calculated for each MC4R mutant cAMP production curve considering total peak area above the baseline calculated as the average signal for mock pcDNA3.1(+)-transfected cells. For data normalization, the AUC from mock transfected cells was set as 0 and the AUC from WT MC4R was set as 100%. Results are from 3-8 independent experiments.

#### ERK1/2 phosphorylation immunoblotting

For time-course ERK1/2 phosphorylation experiments, 1.5 × 10^5^ HEK293 cells were seeded in a poly-D-lysine coated 24-well plate and transfected the next day with 250 ng of MC4R WT or mutant constructs. Cells were serum starved overnight and stimulated for indicated periods of time (3, 5, 10, 15 and 30 min) with NDP-α-MSH (1 μM) before harvesting for immunoblotting. For experiments on knockout cells, 1.0 × 10^5^ Parental HEK293, 1.2 × 10^5^ ARRB1/2 KO HEK293 or 1.2 × 10^5^ GNAS KO HEK293 cells were seeded in a poly-D-lysine coated 24-well plate and transfected the next day with 250 ng of MC4R WT or mutant constructs. Cells were serum starved overnight and stimulated with NDP-α-MSH (1 μM) for 3 min. When appropriate, 100 μM Barbadin, 80 μM dynasore, 10 μM H89 (Sigma-Aldrich, B1427) or DMSO vehicle were added to the well 30 minutes before NDP-α-MSH stimulation. For gene knockdown experiments using siRNA, 20,000 HEK293 cells were transfected with 10 nM siBARR1, siBARR2, siGNAS or siControl (non-targeting siRNA) 24 hours prior to MC4R plasmids transfection, using Lipofectamine RNAiMAX (Invitrogen, 13778150). After NDP-α-MSH (1 μM) stimulation for the indicated periods of time, cells were washed once with PBS and lysed in radio-immunoprecipitation assay buffer (RIPA) (Sigma, R0278) supplemented with protease and phosphatase inhibitors (Roche cOmplete, Mini Protease Inhibitor Cocktail, 11836153001; Roche PhosSTOP, PHOSS-RO). After being harvested from the wells and centrifuged at 14,000 rpm for 15 min, the samples were prepared for electrophoresis (resuspended in 1x Bolt LDS sample buffer (Thermo, B0007) and 1x Bolt reducing agent (Thermo, B0009) and heated for 10 min at 85°C). Equal amounts of protein were loaded and protein electrophoresis was performed using Bolt 4%–12% Bis-Tris Plus gels (Thermo, NW04125BOX) and transferred onto nitrocellulose membrane using an iBLOT (Thermo, IB301001). After blocking with 5% bovine serum albumin (BSA) solution in Tris-buffered saline (TBS) supplemented with 0.1% Tween 20 (TBS-T) for 1 hour at room temperature, membranes were probed overnight at 4^ο^C using a Rabbit anti-p44/42 mitogen-activated protein kinase (MAPK) (extracellular signal–regulated kinases, Erk1/2) (137F5) at 1:1000 dilution (Cell Signaling Technology, 4695), a Rabbit anti-Phospho-p44/42 MAPK (Erk1/2) (Thr202/Tyr204) at 1:1000 dilution (Cell Signaling Technology, 9101), a Goat anti-GNAS at 1:500 dilution (Thermo, PA5-19315), a Rabbit anti-β-arrestin-1/2 (D24H9) at 1:1000 dilution (Abcam, ab129002), all prepared in the blocking buffer. Blots were washed three times with TBS-T for 10 min at room temperature and subsequently incubated with secondary antibody, Goat anti-rabbit IgG-HRP (Dako, P0448) or Donkey anti-goat IgG-HRP (Santa Cruz, sc-2056) diluted 1:2500 in 2% BSA in TBS-T for 1 hour at room temperature. Bands were developed using enhanced chemiluminescence (ECL) substrate (Promega, W1015) and images were captured with an ImageQuant LAS 4000 (GE Healthcare). The band intensity of western blots was quantified using FIJI. For data normalization, unstimulated WT MC4R readouts were set as baseline (0), and maximum WT MC4R ERK1/2 phosphorylation upon agonist stimulation was set as 100%. Time-course ERK1/2 phosphorylation data was plotted as [pERK1/2]/[ERK1/2]. Results are from 3-14 independent experiments.

#### Structural mapping of MC4R mutants

In order to determine which receptor regions were affected by natural variation, we mapped MC4R mutants into the recently crystallized structure of MC4R in complex with SHU9119 (PDB: 6W25) (Yu et al., 2020). We also generated homodimer models for two previously proposed receptor interfaces formed by receptor – receptor interactions between helices TM-1 / TM-7 and TM-4 / TM-5 (Heyder et al., 2019). To do so, we used the starting crystal structure of MC4R to generate dimers based on the crystallized CXCR4 (PDB: 3OE0) (Wu et al., 2010) and kappa-opioid receptors (PDB: 4DJH) (Wu et al., 2012) respectively. To optimize such models, the resulting systems were embedded in a hydrated (water phase containing 0.15M NaCl) 110x110Å POPC bilayer and equilibrated using the six-step Namd protocol for membrane proteins provided by the CHARMM-GUI system builder with the CHARMM36m forcefield (Wu et al., 2014; Huang et al., 2017). All resulting models where visualized and analyzed with VMD 1.9.3 software (Humphrey et al., 1996), which was also used to select MC4R mutants in the receptor – receptor interface as those within a 4Å distance of the opposite dimerization partner.

#### MC4R homodimerization assay

MC4R dimerization was monitored using NanoBiT protein:protein interaction assay (Promega®, M2014). MC4R WT was cloned into both pBiT1.1-C [TK/LgBiT] and pBiT2.1-C [TK/SmBiT] vectors and MC4R mutants were cloned into pBiT1.1-C [TK/LgBiT] vector. Assays were performed in HEK293 cells seeded in poly-D-lysine-coated, white 96-well plates (40,000 cells/well) transiently transfected with 50 ng/well of each of the two constructs as follow: MC4R WT-SmBiT/MC4R WT-LgBiT; MC4R WT-SmBiT/MC4R mutant-LgBiT. For the negative control, MC4R WT-SmBiT construct was substituted with NanoBiT negative control vector (HaloTag-SmBiT). Positive control consisted of SmBiT-PRKACA and LgBiT-PRKAR2A vectors and was added to every experiment. Following transfection, cells were maintained overnight in cell culture medium as specified previously. The next day, protein-protein interaction assay was performed as described above. The area under the curve (AUC) was calculated for each condition using the average value for the negative control as the baseline and the total peak of each curve. For data normalization, the AUC from the negative control was set as 0 and the AUC from MC4R WT-SmBiT/MC4R WT-LgBiT was set as 100%. Results are from 4-7 independent experiments.

#### Determination of signaling bias using stable reporter cell lines

Stably expressing cAMP and β-arrrestin-2 reporter cell lines used for the characterization of MC4R ligands were generated by Ca^2+^ precipitation transfection of naive HEK293 cells with respective cDNA constructs containing eukaryotic antibiotic resistance genes and subsequent selection with respective antibiotics (Figure S8). Approximately 4 × 10^6^ cells were seeded in 15 cm^2^ dish and cultured overnight. Cell culture media was aspirated and replaced with half of the original volume of fresh media. For each transfection, total of 40 μg of cDNA (2 × 20 μg in case of cAMP reporter cell line) was diluted in 420 μl TE buffer and 60 μl 2 M CaCl_2_ was added. DNA was precipitated by adding dropwise into 2x HEPES-buffered saline while gentle swirling the tube. Following 45 min incubation at ambient temperature, the precipitated DNA was added to gently to the cells. For the cAMP reporter cell line, MC4R WT cDNA construct in pcDNA3.1(+) vector and pGloSensor-20F cAMP reporter plasmid (Promega, E1171) were transfected. For β-arrrestin-2 reporter cell line, cDNA fragments encoding human MC4R WT C-terminally tagged with LgBiT (larger fragment of split NanoLuciferase) and human β-arrestin-2 N-terminally tagged with SmBiT were cloned into a NanoBiT CMV MCS BiBiT vector with bi-directional promoter (Promega, CS1603B32). The optimal concentration of antibiotics used for selecting resistant cells were determined in killing curve experiments. Day after transfection, cell culture media was replaced with respective selection media containing 250 μg/ml Geneticin (G418, Sigma-Aldrich, G8168) and 150 μg/ml Hygromycin B (Invitrogen, 10687010) or 5 ng / ml Blasticidin S (GIBCO, A1113903), for the selection of MC4R WT-cAMP GloSensor and MC4R WT-β-arrestin-2 BiBiT reporter cell lines, respectively. Medium was changed approximately every 2 days to ensure there was no excess of dead cells, until single cells remained in the dish and clonal growth of foci was observed. Once foci became visible macroscopically, cell culture medium was removed, cells washed with PBS and cells from at least 10 individual foci were lifted by scraping with a pipette tip and transferred to a 24-well plate where they were re-suspended in 1 mL of respective selection media. Clones which survived the selection and transfer were further propagated into bigger cell culture flasks. Obtained clones were validated for signaling efficiency using respective cAMP GloSensor or β-arrestin-2 recruitment protocols as outlined earlier. Clones responding to NDP-α-MSH stimulation with the best signal to background ratio were selected for further studies.

Recombinant endogenous MC4R agonists, α-, β- and γ-MSH, a set of synthetic peptides and a small molecule agonist, tetrahydroisoquinoline (THIQ) were selected to for functional characterization using both stable reporter cell lines (Figure S8). Each ligand was tested at three concentrations; 0.1 nM, 10 nM and 1 μM, in duplicates in each reporter cell line. Efficacy of each ligand response was quantified as AUC with vehicle-treated reporter cells as baseline and compared to α-MSH response in respective assay plate (referred as % of α-MSH activity at the same concentration of the ligand). Mean relative values from 4-5 independent experiment were combined and data for 10 nM efficacy in both assays are presented in scatterplot diagram.

### Quantification and statistical analysis

Results were analyzed using GraphPad Prism 8 (Graph Pad Software). The difference of mutant MC4R with wild-type was estimated and tested using an unpaired t test with Welch’s correction assigning a value of 100% for WT. When more than two independent variables were analyzed simultaneously, statistical significance was determined by two-way ANOVA followed by Dunnet’s multiple comparison test. Studies in cellular models are from at least 3 independent experiments. All p values reported in this manuscript are from 2-sided statistical tests. A p < 0.05 was considered statistically significant. In figures, statistical significance is represented as ^∗^p < 0.05, ^∗∗^p < 0.01 and ^∗∗∗^p < 0.001.

## Supporting Citations

The following reference appears in the supplemental information: Isberg et al. (2015).

## Supplemental information

Document S1. Figures S1–S8 and Data S1

Table S1. Summary of functional characterization of MC4R mutants in cells, related to Figure 1 MC4R mutations that do and do not affect PM localisation are included; GPCRdb generic residue numbers are noted according to Isberg et al. (2015). N-/C-term, N-/C-terminal domain; TM, transmembrane domain; ELC/ICL, extracellular/intracellular loop; PM, plasma membrane; EE, early endosomes; SD, standard deviation; n, number of replicates; effect defined by the mean for a mutation statistically significantly different from WT; GoF, gain-of-function; (-) no significant change / WT-like; LoF, loss-of-function; statistical difference between means assessed in unpaired single-sample t test with Welch’s correction (^∗^p < 0.05; ^∗∗^p < 0.01; ^∗∗∗^p < 0.001); light gray shading, values not determined.

Table S2. Phenotypes of *MC4R* variant carriers, related to Figure 1 Obese patients from the GOOS cohort with mutations in *MC4R* that were studied here, are shown. Some had multiple attendances. M-male; F-female; (-) Data not available. Height and BMI SDS were calculated up to age 22. IGT, impaired glucose tolerance; BMI, body mass index; SDS, standard deviation score; Type 2 DM, type 2 diabetes mellitus; SBP, systolic blood pressure; DBP, diastolic blood pressure are measured in millimeters of mercury (mmHg). Normal range for fasting insulin in this assay = 0-60 pmol/l. ^ˆ^Also carries *MC4R* E61K (het). ^∼^Also carries *MC4R* T112NfsX11 (het). ^∼∼^Also carries M200del. ^#^Homozygous, ^∗^6 months post sleeve gastrectomy.

Document S2. Article plus supplemental information
