# Supplementary material for: Human MC4R variants affect endocytosis, trafficking and dimerization revealing multiple cellular mechanisms involved in weight regulation
Source: Cell Rep. 2021 Mar 23;34(12):108862. doi: 10.1016/j.celrep.2021.108862 (PMC7994375; doi:10.1016/j.celrep.2021.108862)
Supplement: Document S1. Figures S1–S8 and Data S1 [file mmc1.pdf]

Cell Reports, Volume *34*

Supplemental information

Human MC4R variants affect endocytosis, trafficking and dimerization revealing multiple cellular mechanisms involved in weight regulation

Bas Brouwers, Edson Mendes de Oliveira, Maria Marti-Solano, Fabiola B.F. Monteiro, Suli-Anne Laurin, Julia M. Keogh, Elana Henning, Rebecca Bounds, Carole A. Daly, Shane Houston, Vikram Ayinampudi, Natalia Wasiluk, David Clarke, Bianca Plouffe, Michel Bouvier, M. Madan Babu, I. Sadaf Farooqi, and Jacek Mokrosiński

SUPPLEMENTAL INFORMATION

Figure S1

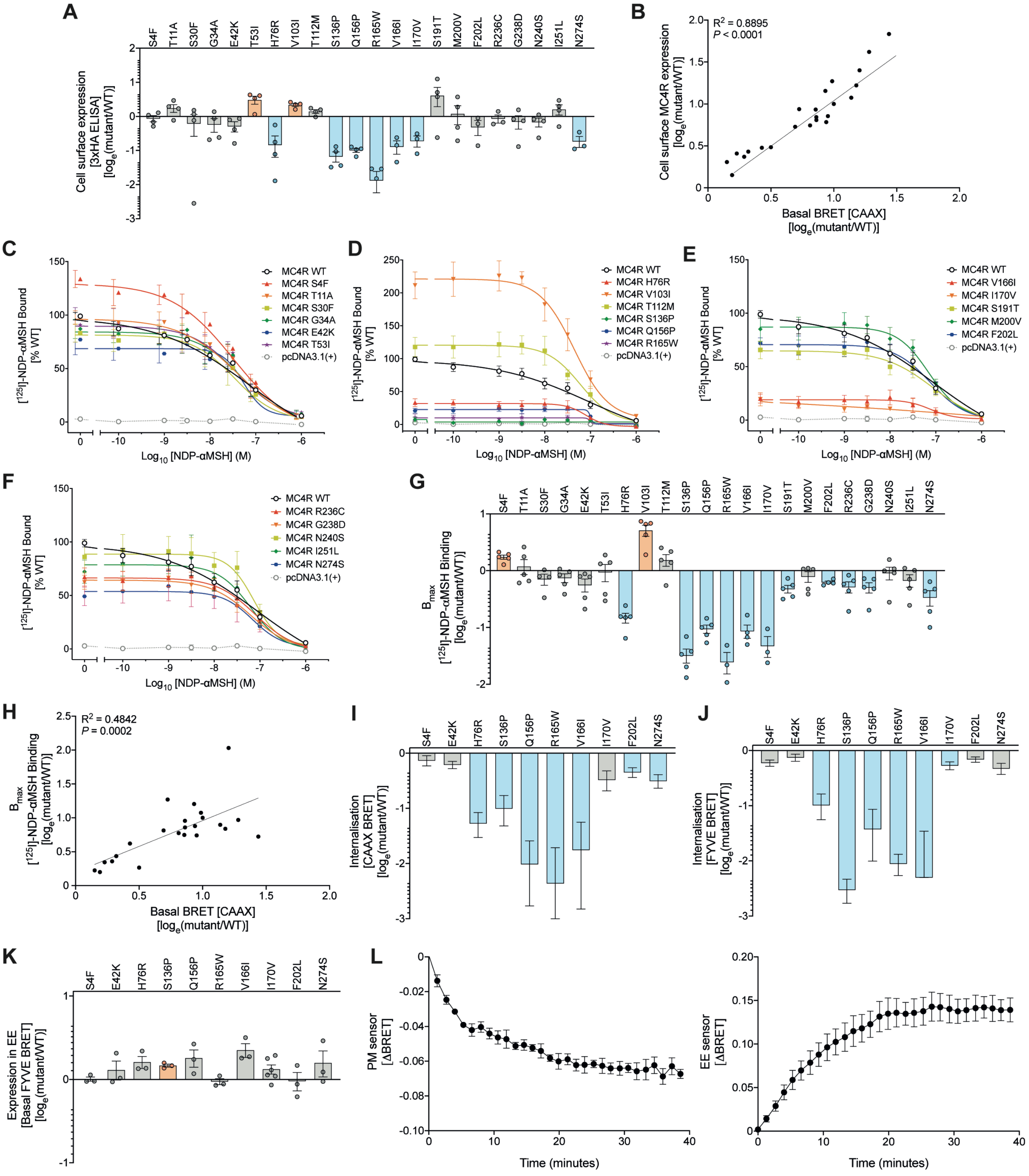

Figure S1. Cellular localization and binding of MC4R mutants. A, Cell surface expression of WT and mutant MC4Rs measured by enzyme-linked immunosorbent assay (ELISA). **B**, Pearson correlation between receptor number at the plasma membrane quantified using the PM sensor (basal ebBRET) and cell surface ELISA. **C-F** Competitive binding assays. HEK293 cells transiently expressing WT or mutant MC4R were incubated with [^{125}I]-NDP-α-MSH in the presence of increasing concentrations of unlabelled ligand. **G**, Apparent maximal specific binding (B_{max}). **H**, Pearson correlation between receptor number at the plasma membrane quantified using the PM sensor (basal ebBRET) and B_{max}. **I**, Agonist-induced internalisation as quantified using the PM sensor, which was corrected for basal ebBRET [rGFP-CAAX]. **J**, Agonist-induced internalisation quantified by means of the EE sensor, which was corrected for basal ebBRET [rGFP-CAAX]. **K**, Basal expression of receptor mutants in EEs (basal ebBRET using the EE sensor). **L**, Kinetics experiment showing NDP-α-MSH dependent MC4R endocytosis, using the PM sensor (left panel) and the EE sensor (right panel). ΔBRET is defined as $BRET_{stimulated}$-$BRET_{vehicle}$. **A**, **G**, **I-K**, Data are expressed as log_e(mutant/WT) and plotted as mean $\pm$ standard error of 3-6 independent experiments. In panels, **A**, **G**, **I**, **J**, **K** mutants classified as Gain-of-Function (GoF) (orange), Loss-of-Function (LoF) (blue) or WT-like (grey) based on statistically significant differences between WT and mutant (unpaired *t*-test with Welch's correction; $p < 0.05$). Related to Figure 1.

Figure S2

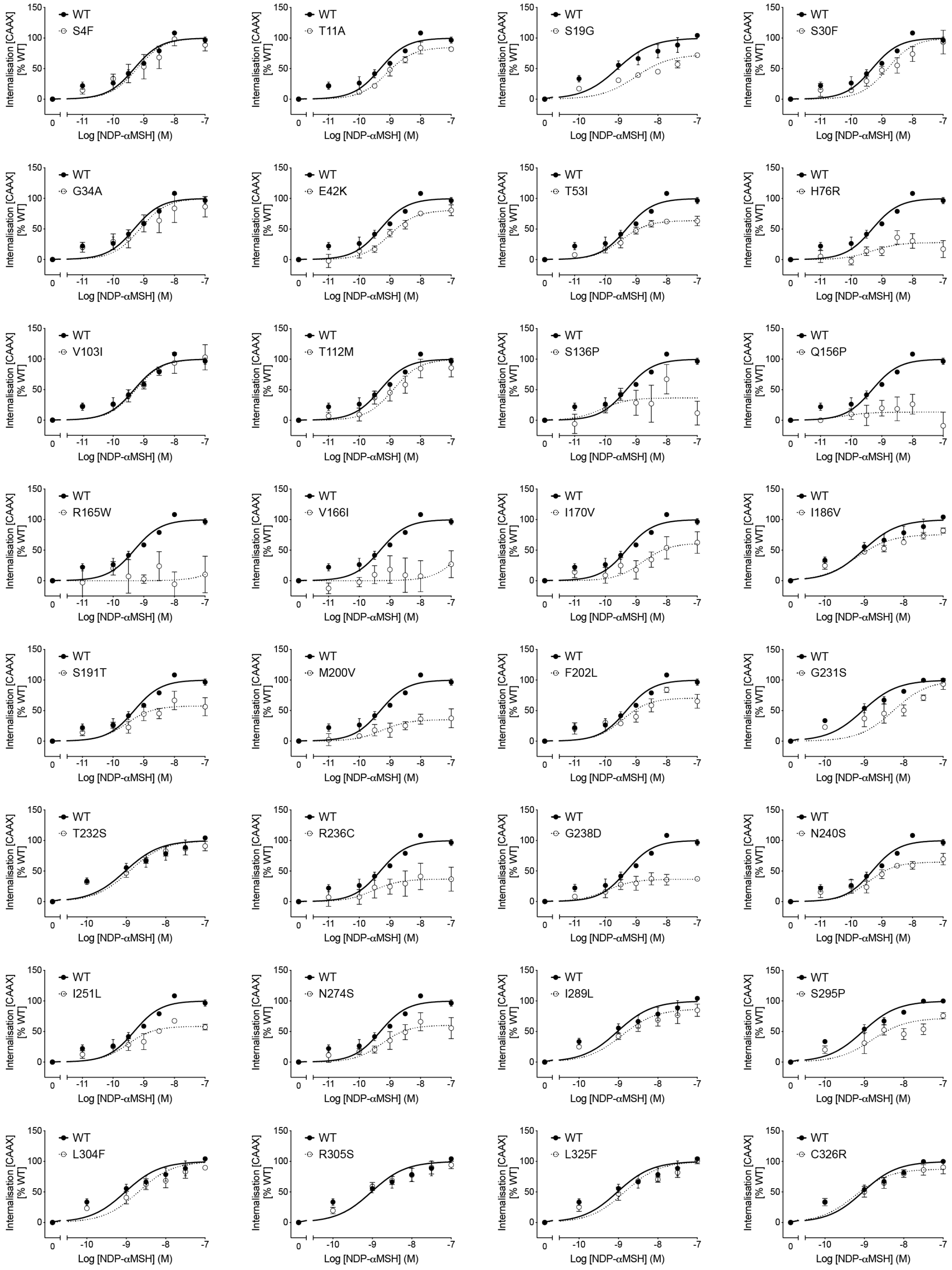
Internalisation [CAAX] [% WT]
Log [NDP-αMSH] (M)
WT
S4F
T11A
S19G
S30F
G34A
E42K
T53I
H76R
V103I
T112M
S136P
Q156P
R165W
V166I
I170V
I186V
S191T
M200V
F202L
G231S
T232S
R236C
G238D
N240S
I251L
N274S
I289L
S295P
L304F
R305S
L325F
C326R

Figure S2. Impaired endocytosis of mutants in MC4R. Agonist (NDP-α-MSH)-induced receptor endocytosis of wild-type MC4R and mutants quantified by ebBRET, using the PM sensor. Internalisation is expressed as percentage of the maximal ΔebBRET obtained with wild-type (WT) receptor and is corrected for basal receptor presence at the plasma membrane as measured by the PM sensor (see Methods). Data are plotted as mean ± standard error of 3 independent experiments. Related to Figure 1.

Figure S3

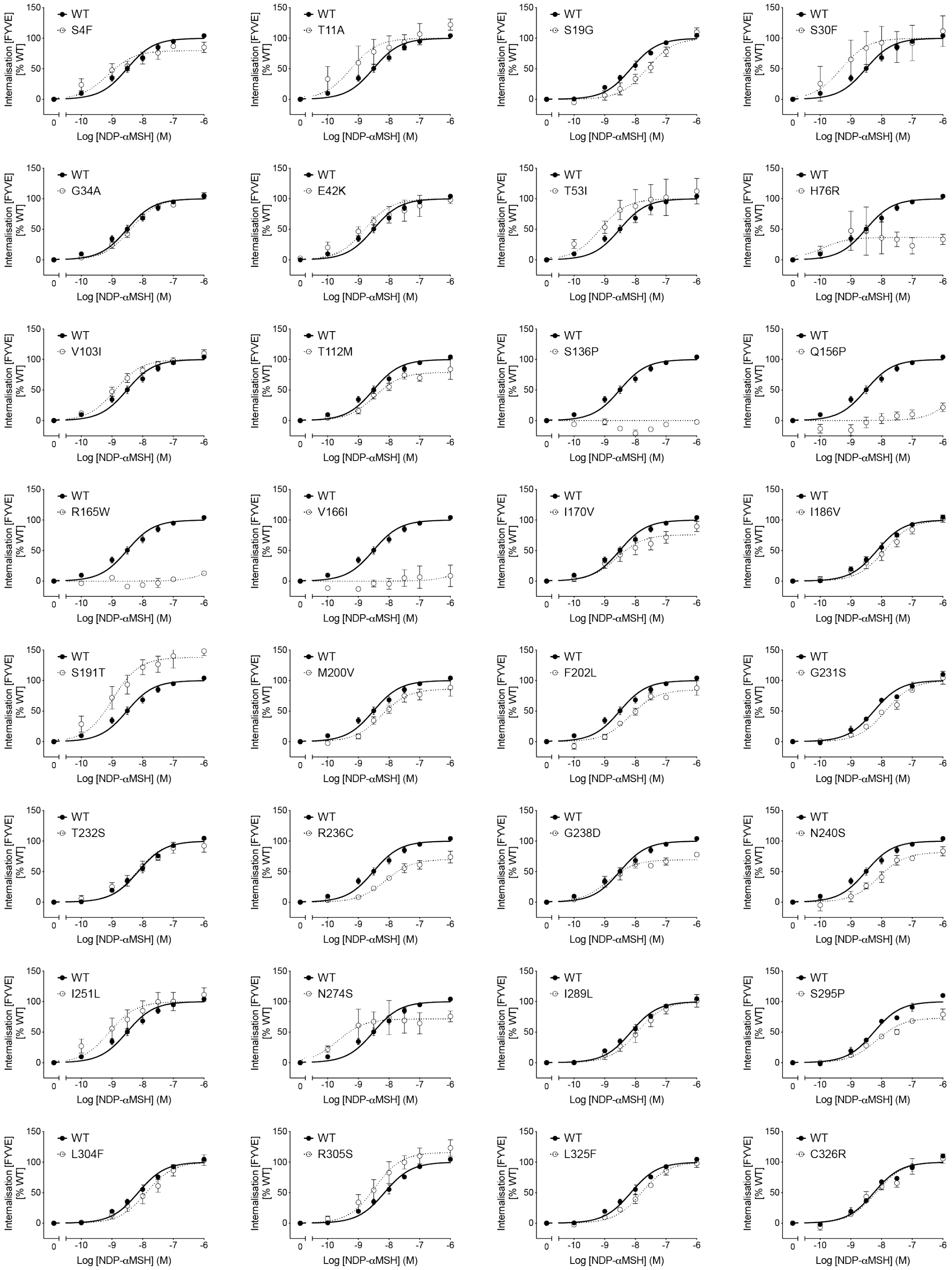
Internalisation [FYVE] [% WT]
Log [NDP-αMSH] (M)
WT
S4F
T11A
S19G
S30F
G34A
E42K
T53I
H76R
V103I
T112M
S136P
Q156P
R165W
V166I
I170V
I186V
S191T
M200V
F202L
G231S
T232S
R236C
G238D
N240S
I251L
N274S
I289L
S295P
L304F
R305S
L325F
C326R

Figure S3. Impaired trafficking of mutants in MC4R into early endosomes. Agonist (NDP-α-MSH)-induced endocytosis of wild-type (WT) MC4R and mutants quantified by ebBRET, using the EE sensor. Internalisation is expressed as percentage of the maximal ΔebBRET obtained with wild-type (WT) receptor and is corrected for basal receptor presence at the plasma membrane as measured by the PM sensor (see Methods). Data are plotted as mean ± standard error of 3-6 independent experiments. Related to Figure 1.

Figure S4

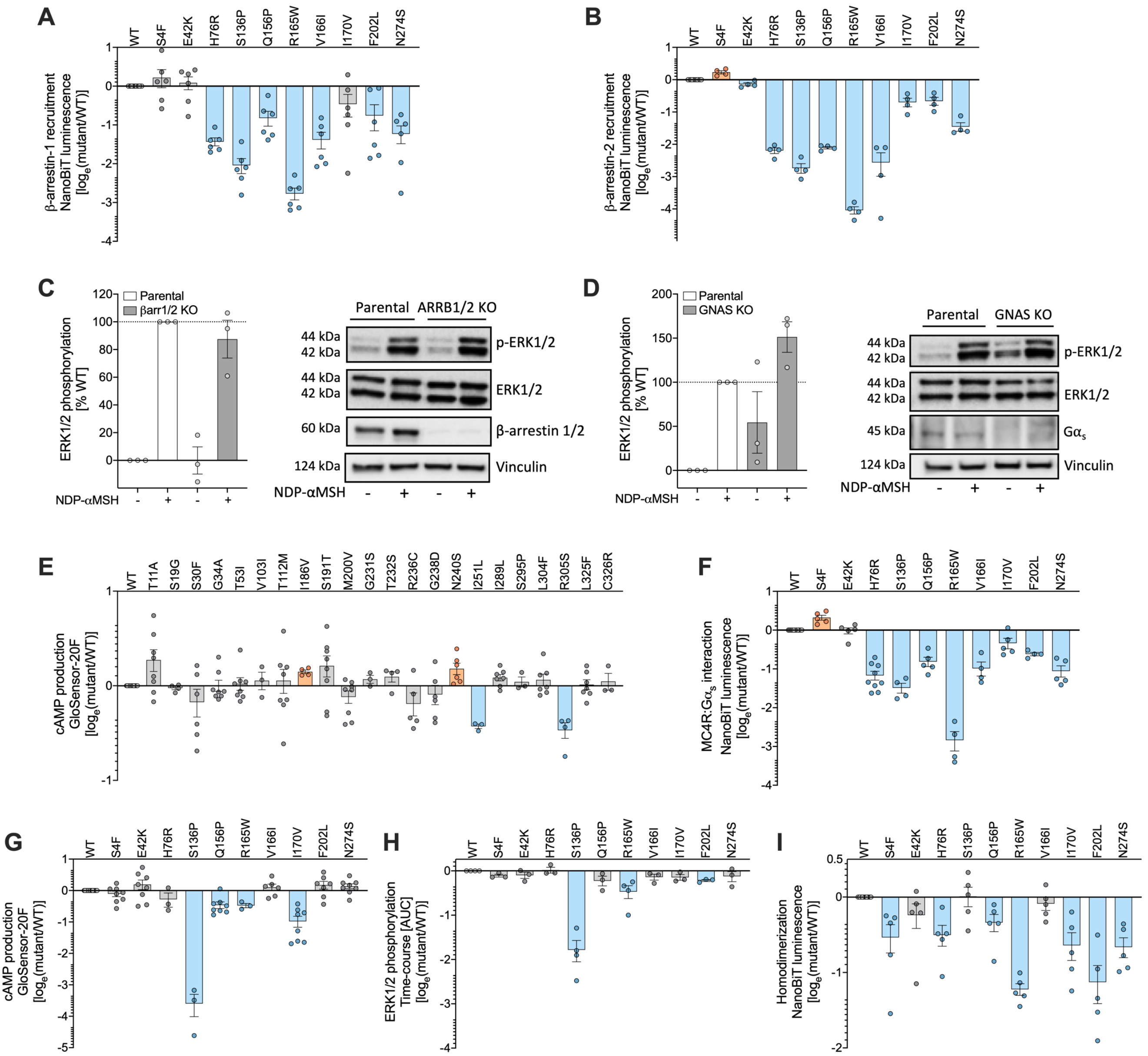

A
β-arrestin-1 recruitment NanoBiT luminescence [log$_e$(mutant/WT)]
WT S4F E42K H76R S136P Q156P R165W V166I I170V F202L N274S
B
β-arrestin-2 recruitment NanoBiT luminescence [log$_e$(mutant/WT)]
C
ERK1/2 phosphorylation [% WT]
Parental
βarr1/2 KO
NDP-αMSH
Parental ARRB1/2 KO
44 kDa 42 kDa p-ERK1/2
44 kDa 42 kDa ERK1/2
60 kDa β-arrestin 1/2
124 kDa Vinculin
D
Parental
GNAS KO
45 kDa Gα$_s$
E
cAMP production GloSensor-20F [log$_e$(mutant/WT)]
WT T11A S19G S30F G34A T53I V103I T112M I186V S191T M200V G231S T232S R236C G238D N240S I251L I289L S295P L304F R305S L325F C326R
F
MC4R:Gα$_s$ interaction NanoBiT luminescence [log$_e$(mutant/WT)]
G
cAMP production GloSensor-20F [log$_e$(mutant/WT)]
H
ERK1/2 phosphorylation Time-course [AUC] [log$_e$(mutant/WT)]
I
Homodimerization NanoBiT luminescence [log$_e$(mutant/WT)]

Figure S4. MC4R mutants impair G-protein dependent and G-protein independent signaling. **A-B**, Impact of MC4R mutants which decrease PM expression on (**A**) β-arrestin-1 and (**B**) β-arrestin-2 recruitment. **C-D**, NDP-α-MSH-dependent ERK1/2 phosphorylation in (**C**) ARRB1/2 and (**D**) GNAS knockout cells. Representative immunoblots shown were probed for total ERK1/2, phosphorylated ERK1/2 [p-ERK1/2], β-arrestin-1/2 or $G\alpha_s$, and vinculin as an additional loading control. **E**, cAMP production for mutants that do not negatively affect receptor number at the plasma membrane. **F**, MC4R : $G\alpha_s$ interaction and **G**, cAMP production upon receptor stimulation with 1 μM NDP-α-MSH. **H**, NDP-α-MSH mediated ERK1/2 phosphorylation for MC4R mutants. **i**, MC4R homodimerization quantified using NanoBiT. All data in panels **A-B** and **F-I** are expressed as $\log_e$(mutant/WT) and plotted as mean ± standard error of 3-9 independent experiments and mutants classified as Gain-of-Function (GoF) (orange), Loss-of-Function (LoF) (blue) or WT-like (grey) based on statistically significant differences between WT and mutant (unpaired *t*-test with Welch's correction; $p < 0.05$). **C-D**, Data are expressed as a percentage of maximal control response [% WT] and plotted as mean ± standard error from 3-14 independent experiments. Statistical significance was determined by two-way ANOVA and Dunnet's multiple comparison test and no difference was observed when NDP-α-MSH-stimulated MC4R WT [KO cell line] was compared to the parental line). Related to Figures 2 and 4.

Figure S5

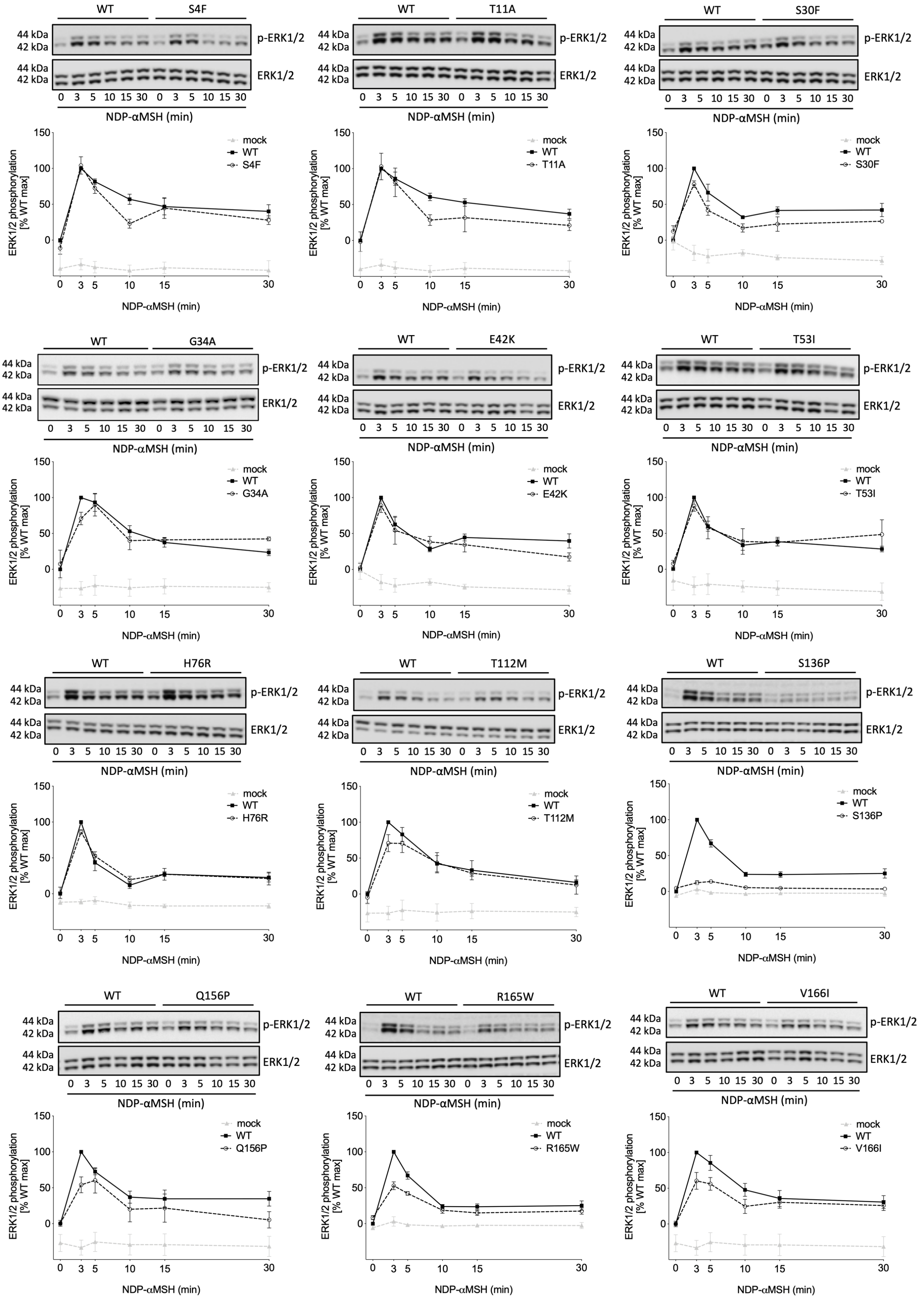
WT
S4F
T11A
S30F
G34A
E42K
T53I
H76R
T112M
S136P
Q156P
R165W
V166I
44 kDa
42 kDa
p-ERK1/2
ERK1/2
0 3 5 10 15 30 0 3 5 10 15 30
NDP-αMSH (min)
ERK1/2 phosphorylation [% WT max]
150
100
50
0
0 3 5 10 15 30
mock

Figure S5. Effect of MC4R mutants on ERK1/2 phosphorylation. Time course experiment showing ERK1/2 phosphorylation downstream of MC4R upon stimulation with 1 μM NDP-αMSH (MC4R S4F to MC4R V166I mutants). Representative blots for p-ERK1/2 and ERK1/2 are shown. Each graph represents mean ± standard error from 3-5 independent experiments. Statistics were performed on area under the curve (AUC) quantifications of each graph (Methods). Related to Figure 4.

Figure S6

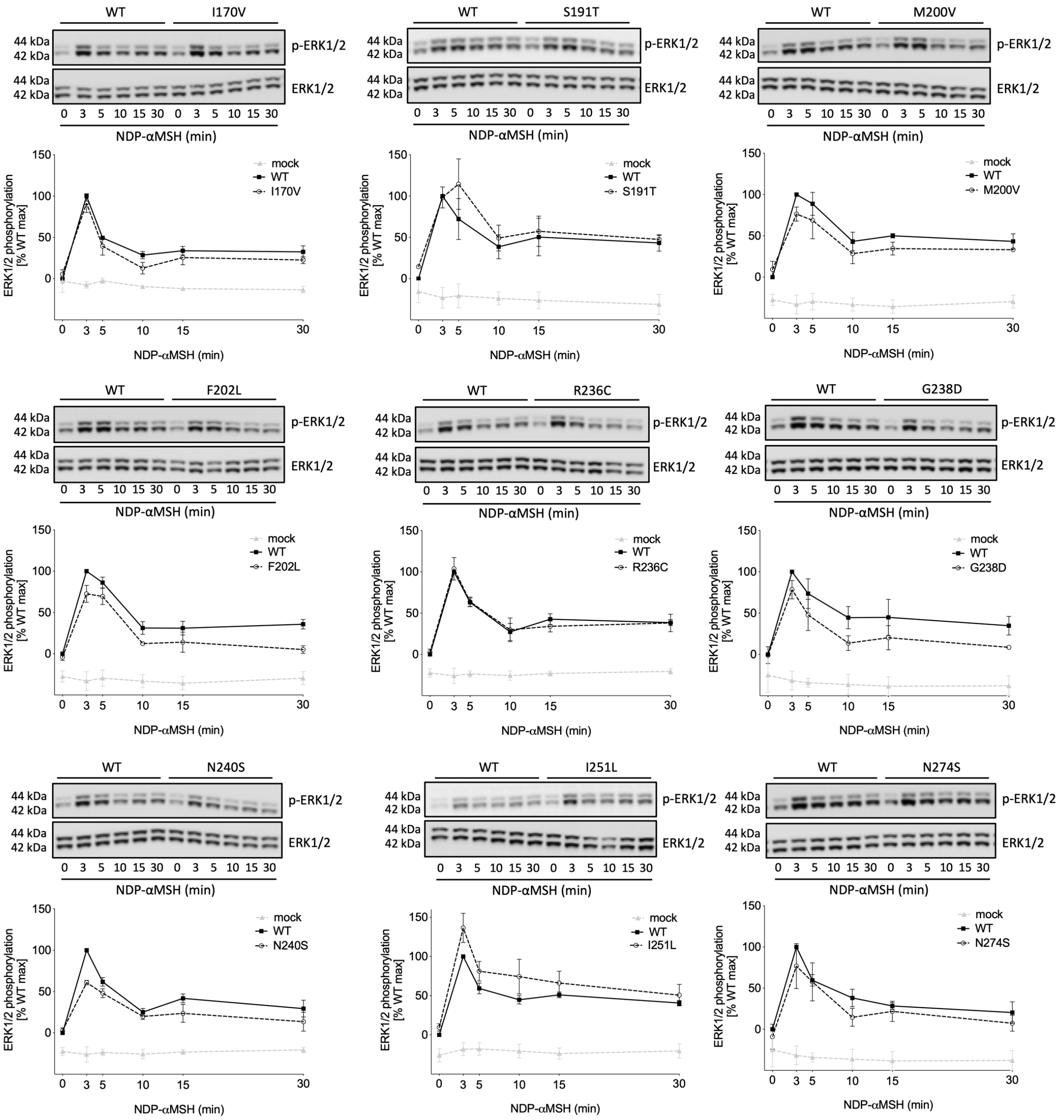
WT
I170V
S191T
M200V
F202L
R236C
G238D
N240S
I251L
N274S
44 kDa
42 kDa
p-ERK1/2
ERK1/2
0 3 5 10 15 30 0 3 5 10 15 30
NDP-αMSH (min)
mock
ERK1/2 phosphorylation [% WT max]
150
100
50
0
0 3 5 10 15 30

Figure S6. Effect of MC4R mutants on ERK1/2 phosphorylation. Time course experiment showing ERK1/2 phosphorylation downstream of MC4R upon stimulation with 1 μM NDP-αMSH (MC4R I170V to MC4R N274S mutants). Representative blots for p-ERK1/2 and ERK1/2 are shown. Each graph represents mean ± standard error from 3-5 independent experiments. Statistics were performed on area under the curve (AUC) quantifications of each graph (Methods). Related to Figure 4.

Figure S7

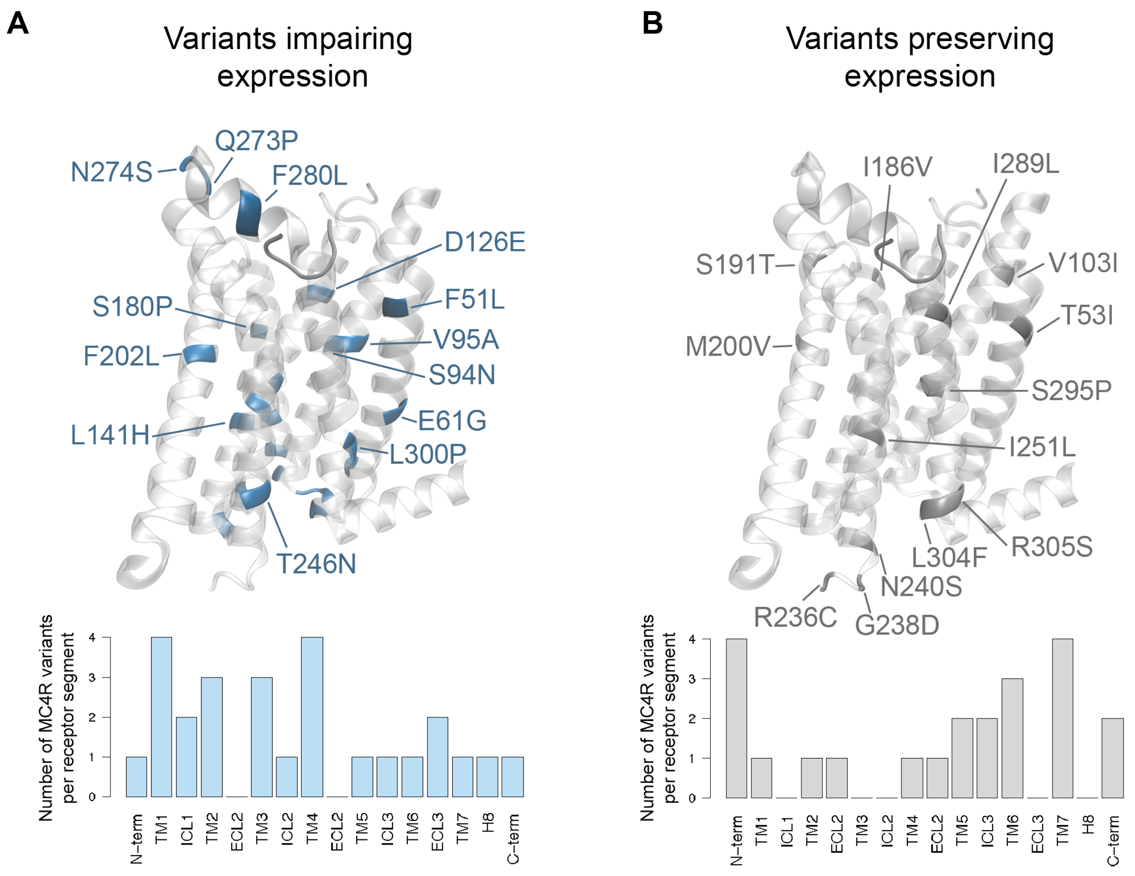

Figure S7. Structural mapping of MC4R mutants that do/do not affect plasma membrane expression. Receptor mutants resulting in (**A**) reduced (≤ 85% of WT) or (blue) (**B**) normal plasma membrane expression (grey) are shown mapped onto a cartoon representation of the crystallised MC4R receptor structure (PDB ID: 6W25). The crystallised ligand, SHU9119, is shown as cartoon in dark grey. Mapping the abundance of both classes of mutants in different receptor structural segments reveals a different distribution of mutants disrupting or preserving MC4R expression. Related to Figure 1.

Figure S8

A

Peptide	Nmod	Sequence	Cmod
MSH tetrapeptide	H	H F R W	OH
		1 2 3 4 5 6 7 8 9 10 11 12 13	
ACTH (4-10)	H	M E H F R W G	OH
αMSH	Ac	S Y S M E H F R W G K P V	NH_2
Des-acetyl-αMSH	-	S Y S M E H F R W G K P V	NH_2
Diacetyl-αMSH	2xAc	S Y S M E H F R W G K P V	NH_2
DP-αMSH	Ac	S Y S M E H dF R W G K P V	NH_2
NDP-αMSH	Ac	S Y S Nle E H dF R W G K P V	NH_2
H-9220	Ac	C E H dF R W C K P V	NH_2
H-6268	Ac	dR C E H dF R W C	NH_2
LY-2112688	Ac	R C E H dF R W C	NH_2
Setmelanotide	Ac	R C dA H dF R W C	NH_2
Melanotan II	Ac	Nle D H dF R W K	NH_2
SHU9119	Ac	Nle D H d2Nal R W K	NH_2
"MC4R agonist"	-	bA H dF R W E	NH_2
HS024	Ac	C Nle R H d2Nal R W G C	NH_2
JKC-366	-	Mpa Nle R H d2Nal R W G C	NH_2
HP-228	Ac	Nle Q H dF R dW G	NH_2
Nonapeptide-1	H	M P dF R dW F K P V	NH_2
		1 2 3 4 5 6 7 8 9 10 11 12 13 14 15 16 17 18 19 20 21 22	
βMSH	H	A E K K D E G P Y R M E H F R W G S P P K D	OH
VA-βMSH	?	V A A E K K D E G P Y R M E H F R W G S P P K D	OH
monkey βMSH	H	D E G P Y R M E H F R W G S P P K D	OH
porcine βMSH	?	D E G P Y K M E H F R W G S P P K D	?
HS014	As	C E H d2Nal R W G C P P K D	NH_2
JKC-363	-	Mpa E H d2Nal R W G C P P K D	NH_2
JKC-372	-	Mpa E H dF R W C S P P K D	NH_2
		0 1 2 3 4 5 6 7 8 9 10 11 12	
γ_1MSH	H	Y V M G H F R W D R F	NH_2
[Lys^0]- γ_1MSH	?	K Y V M G H F R W D R F	NH_2
γ_2-MSH	H	Y V M G H F R W D R F G	OH
[dTrp^8]-γ_2MSH	H	Y V M G H F R dW D R F G	OH
[Lys^0, Nle^4]- γ_2MSH	Ac	K Y V Nle G H F R W D R F G	NH_2
γ_3MSH	H	Y V M G H F R W D R F G R R N G S S S S G V G G A A Q	OH

B

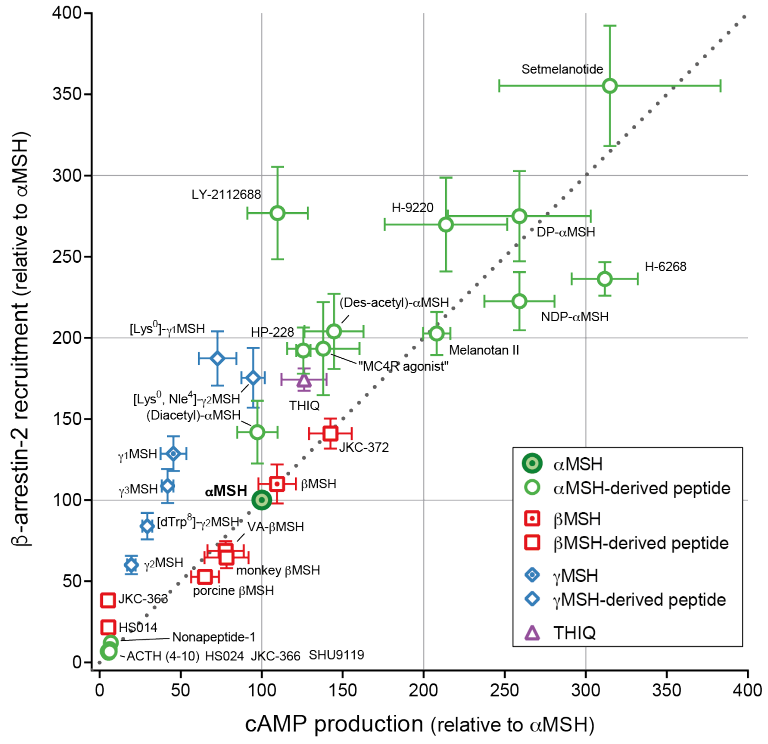

Figure S8. Characterisation of signaling by MC4R ligands and agonists. A, Comparison of the primary amino acid sequence of MC4R peptide ligands and endogenous MC4R agonists (α-MSH, β-MSH and γ_1-MSH) that were tested. D-enantiomers of amino acid residues are indicated with a (d) in front of the symbol, and unnatural amino acids are denoted: Nle, norleucine; d2Nal, 2-naphthylalanine; Mpa, 3-mercaptopropionic acid. N-terminal (Nmod) and C-terminal modifications (Cmod) are indicated as follows: H, N-terminal free amino moiety; OH, unmodified C-terminal carboxyl group; Ac, acetylation; 2xAc, diacetylation, NH3, amidation; (?), not specified. Lines before and after the amino acid residues indicate and span a fragment of the sequence which forms a cyclic peptide stabilized by either a disulphide bond (orange) or a salt bridge (purple). **B**, signalling plot for 32 MC4R ligands (31 peptides and the small molecule agonist; THIQ, tetrahydroisoquinoline). Equimolar efficacy for 10 nM of each ligand determined using stable HEK293 reporter cell lines expressing MC4R WT and cAMP GloSensor™ or β-arrestin-2 split NanoLuciferase BiBiT reporter for the quantification of cAMP production and β-arrestin-2 recruitment, respectively (see Methods). Data presented as mean ± standard error from 4-5 independent experiments normalized to α-MSH signalling efficacy in the respective assay. Related to Figure 2.

Data S1. Uncropped immunoblot images. Related to Figure 3, Figure 4 and Figure S4. For better data visualization, several western blots images showed in this manuscript were cropped for removal of extra lanes in the same blot. It is important to highlight that images collated together in a single image are always originated from the same WB gel and image acquisition, allowing us to compare and analyze the bands in the blots. In the next pages you can find all uncropped western blots images, from all experimental replicates (minimum of 3), performed for this paper. Also, all selected representative WB image used in the paper are identified accordingly.

Data S1

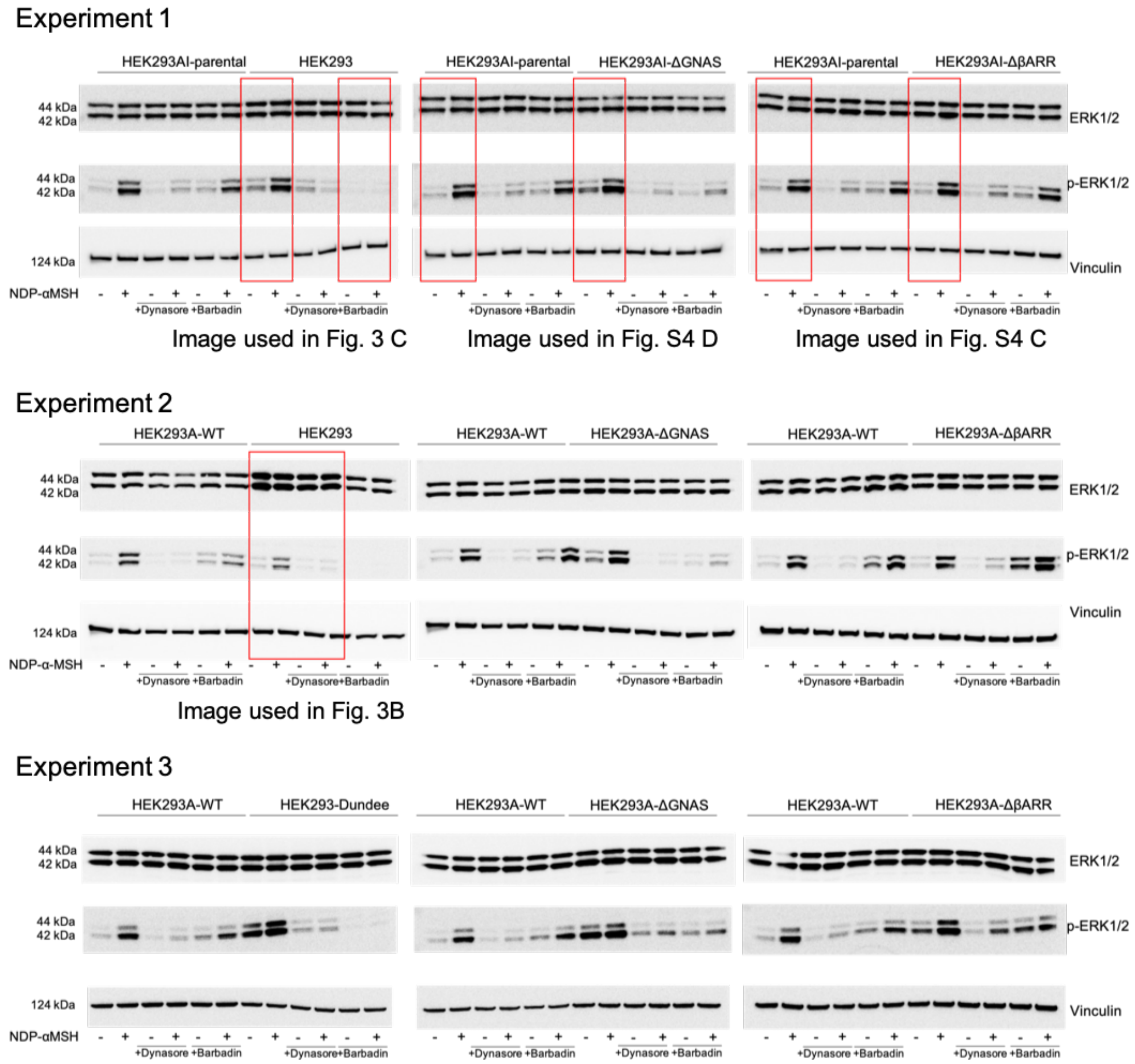

Data S1. Uncropped immunoblot images. ERK1/2 Western Blot using KO cell lines. Images in the red rectangles are the data showed in the corresponding figures. Related to Figure 3 and Figure S4.

Data S1

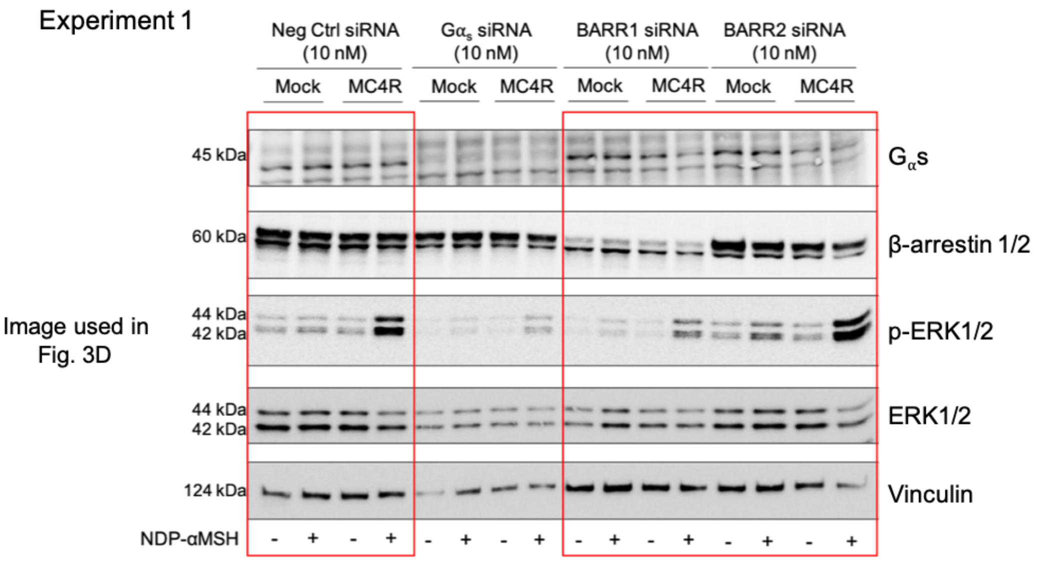
Experiment 1
Neg Ctrl siRNA (10 nM)
Gαs siRNA (10 nM)
BARR1 siRNA (10 nM)
BARR2 siRNA (10 nM)
Mock MC4R Mock MC4R Mock MC4R Mock MC4R
45 kDa
60 kDa
44 kDa
42 kDa
124 kDa
Image used in Fig. 3D
Gαs
β-arrestin 1/2
p-ERK1/2
ERK1/2
Vinculin
NDP-αMSH - + - + - + - + - + - + - + - +

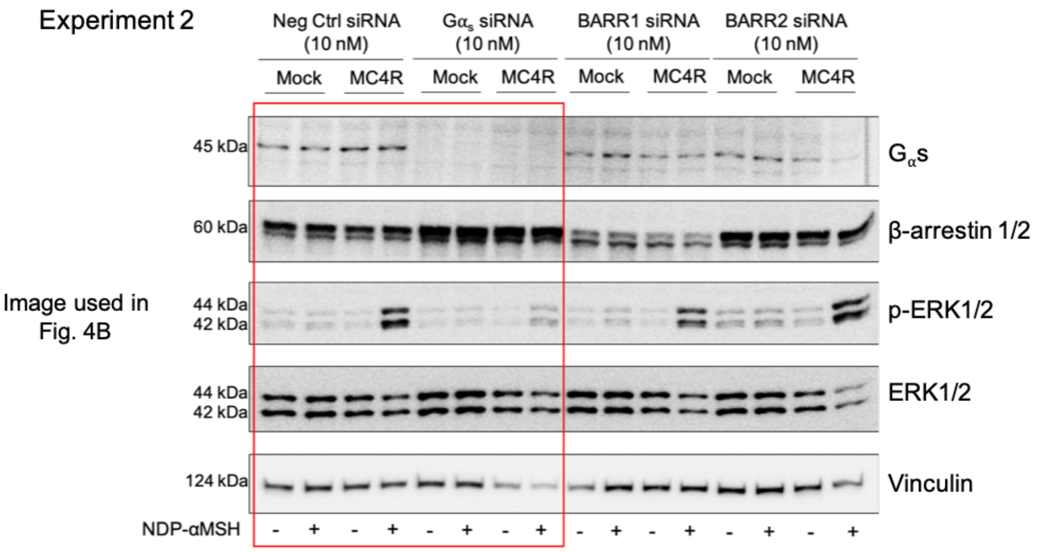
Experiment 2
Neg Ctrl siRNA (10 nM)
Gαs siRNA (10 nM)
BARR1 siRNA (10 nM)
BARR2 siRNA (10 nM)
Mock MC4R Mock MC4R Mock MC4R Mock MC4R
45 kDa
60 kDa
44 kDa
42 kDa
124 kDa
Image used in Fig. 4B
Gαs
β-arrestin 1/2
p-ERK1/2
ERK1/2
Vinculin
NDP-αMSH - + - + - + - + - + - + - + - +

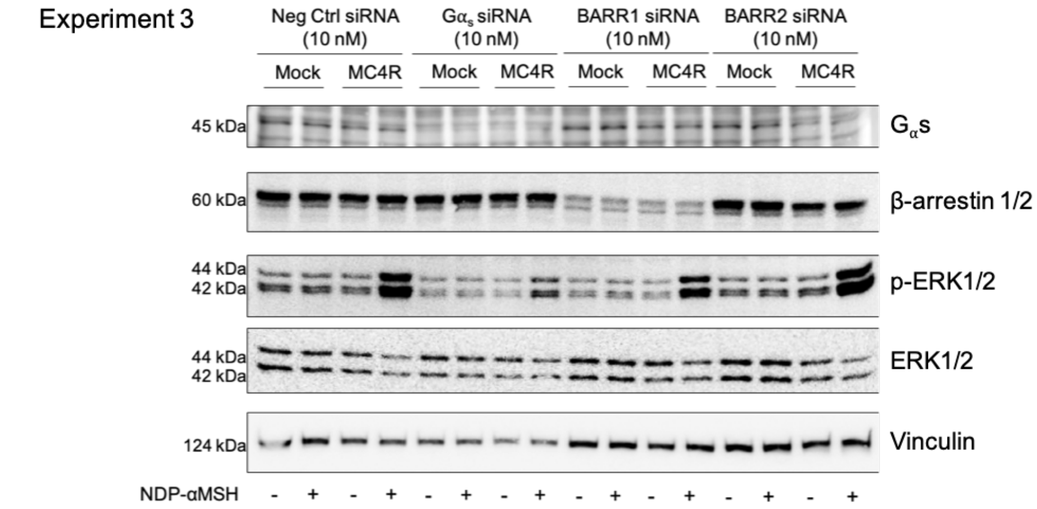
Experiment 3
Neg Ctrl siRNA (10 nM)
Gαs siRNA (10 nM)
BARR1 siRNA (10 nM)
BARR2 siRNA (10 nM)
Mock MC4R Mock MC4R Mock MC4R Mock MC4R
45 kDa
60 kDa
44 kDa
42 kDa
124 kDa
Gαs
β-arrestin 1/2
p-ERK1/2
ERK1/2
Vinculin
NDP-αMSH - + - + - + - + - + - + - + - +

Data S1. Uncropped immunoblot images. ERK1/2 Western Blot using siRNA treatment. Images in the red rectangles are the data showed in the corresponding figures. Related to Figure 3 and Figure 4.

Data S1

Experiment 1

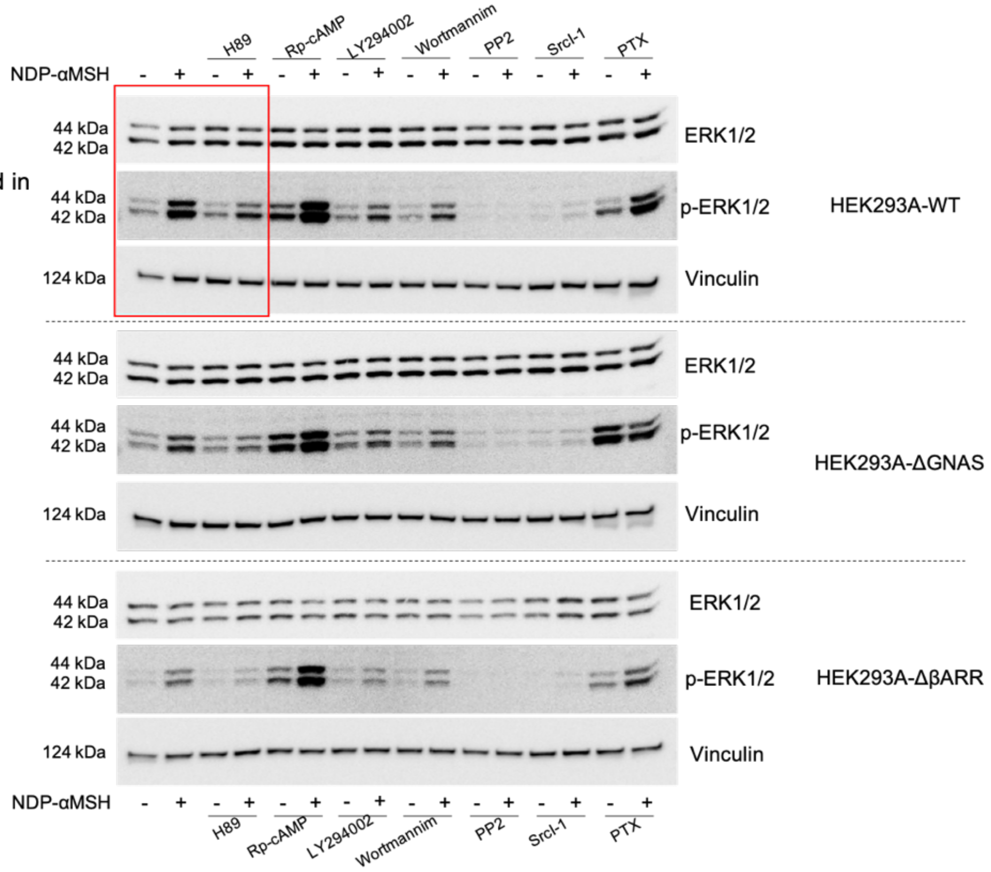

Experiment 2

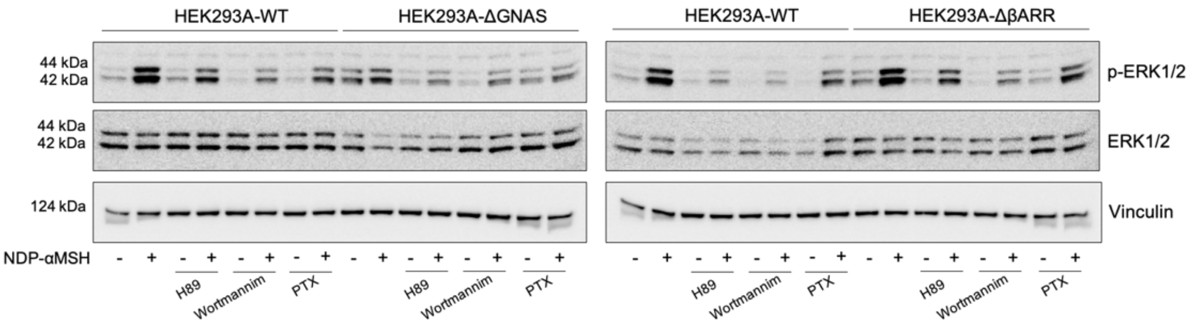

Experiment 3

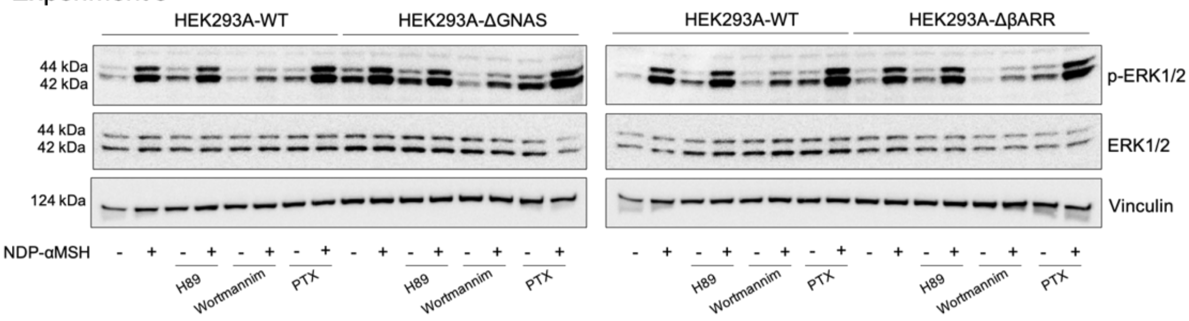

Data S1. Uncropped immunoblot images. ERK1/2 Western Blot using pharmacological inhibitors (H89 treatment). Images in the red rectangles are the data showed in the corresponding figures. Related to Figure 4c.
